# CT-based interpretable delta-radiomics model for risk stratification of pulmonary ground-glass nodules: a multicentre study

**DOI:** 10.1186/s13244-026-02297-2

**Published:** 2026-05-14

**Authors:** Zhen Ye, Yingying Miao, Ping Wang, Qinghe Han, Juntian Gao, Shuang Wang, Ying Ma, Xuezhi Wei, Hanyun Zhang, Kailiang Cheng, Butian Zhang

**Affiliations:** 1 Department of Radiology, China-Japan Union Hospital of Jilin University, Changchun, China; 2 Department of Radiology, Shanghai Sixth People’s Hospital Affiliated to Shanghai Jiao Tong University School of Medicine, Shanghai, China; 3 Department of Radiology, The Second Hospital of Jilin University, Changchun, China; 4 Department of Radiology, Changchun Guowen Hospital, Changchun, China; 5 Department of Radiology, Jinan Third People’s Hospital, Jinan, China

**Keywords:** Lung neoplasms, Ground-glass nodule, Tomography (X-ray computed), Radiomics, Machine learning

## Abstract

**Objectives:**

To develop and externally validate an interpretable fusion model combining multi–time-point CT radiomics with clinical–semantic features to predict invasiveness of pulmonary ground-glass nodules and support three-tier risk stratification.

**Materials and methods:**

In this multicentre retrospective study, patients with pulmonary ground-glass nodules that were resected or managed via CT surveillance with stability (≥ 3 years) were included. Thin-section CT scans at baseline (T0) and follow-up (T1) were used to derive radiomic features at each time point and delta-radiomic features. Four unimodal models (T0 radiomics, T1 radiomics, delta-radiomics, and clinical–semantic) were trained using centre-grouped cross-validation and probability calibration, then fused via stacked logistic regression. Low- and high-risk groups defined by two training-derived and locked probability thresholds were evaluated in an external cohort against clinical and guideline-based models.

**Results:**

The training and external validation cohorts included 358 and 46 patients, respectively. The fusion model achieved an external area under the receiver operating characteristic curve of 0.985 (95% CI: 0.955–1.000) with good calibration. Using training-derived and locked thresholds (0.50 and 0.65), 28.3% of patients were classified as low risk (NPV 100%; 95% CI: 75.3–100.0) and 69.6% as high risk (PPV 93.8%; 95% CI: 79.2–99.2; sensitivity 96.8%; 95% CI: 83.3–99.9). The model reduced false-positive high-risk classifications from 33.3 to 13.3 per 100 non-invasive lesions and showed higher net benefit than comparator models.

**Conclusions:**

An interpretable fusion model enables robust three-tier risk stratification of pulmonary ground-glass nodules and may reduce overdiagnosis and overtreatment in low-dose CT screening programmes.

**Critical relevance statement:**

A calibrated, interpretable fusion model for pulmonary ground-glass nodules enables accurate three-tier risk stratification, reducing false-positive high-risk classifications and supporting safer de-escalation of surveillance in low-risk patients.

**Key Points:**

- An interpretable fusion model combining multi-time-point CT radiomics with clinical-semantic features predicts invasiveness of pulmonary ground-glass nodules.
- The fusion model achieved excellent discrimination (external AUC 0.985) and good calibration, outperforming unimodal radiomics and guideline-based risk models.
- A three-tier risk stratification with calibrated thresholds reduces false-positive high-risk classifications and supports safe de-escalation of surveillance in low-risk patients.

**Graphical Abstract:**

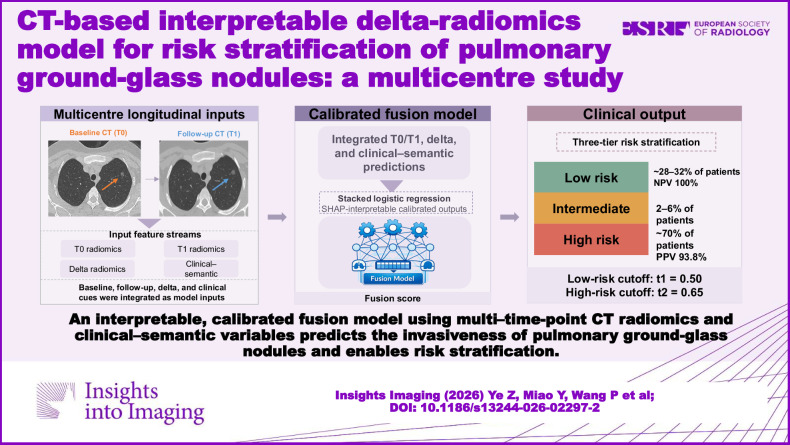

## Introduction

Low-dose CT (LDCT) screening has substantially increased detection of pulmonary ground-glass nodules (GGNs), particularly among never-smoking Asian women [1, 2]. GGNs span a histological continuum from atypical adenomatous hyperplasia (AAH) and adenocarcinoma in situ (AIS) to minimally invasive adenocarcinoma (MIA) and invasive adenocarcinoma (IAC) [3]. This distinction is prognostically important: five-year survival is nearly 100% for AIS/MIA but falls to approximately 70–75% for invasive disease [4, 5]. Yet many GGNs remain stable for years or regress [6, 7]. Early differentiation between invasive and non-invasive GGNs is therefore crucial to avoid overtreatment while ensuring timely intervention.

Guidelines such as the Fleischner recommendations and lung CT screening reporting and data system (Lung-RADS) endorse conservative CT surveillance for small subsolid nodules [6, 8], but their specificity remains limited. Conventional assessments based on two-dimensional morphology and simple size change are imprecise [7]. LDCT screening has high false-positive rates and only modest positive predictive value (PPV) [1, 9], so many indolent GGNs are labelled high risk and undergo invasive procedures or prolonged follow-up [9]. Some apparently stable GGNs later grow or increase in attenuation and are ultimately confirmed as IAC [7], whereas transthoracic needle biopsy has only moderate sensitivity [10]. These limitations highlight the need for more accurate, quantitatively driven risk assessment tools.

Radiomics enables the extraction of quantitative CT features for non-invasive GGN risk stratification and has demonstrated promising performance in both benign–malignant discrimination and invasiveness prediction [11]. Delta-radiomics further captures temporal changes in these features over the course of follow-up [12–14]. However, most existing models rely on single time points or on simple pre-vs-post differences, and they rarely integrate multi–time-point radiomic features with key clinical and radiologic variables in a comprehensive manner. Moreover, these models are typically evaluated primarily by the area under the curve (AUC), with limited assessment of calibration or clinical utility [12–16]. In addition, many radiomics and machine-learning models operate as “black boxes”, which restricts interpretability and hampers clinical adoption [17].

We therefore developed and externally validated an interpretable, probability-calibrated model integrating baseline (T0), follow-up (T1), and delta-radiomics with clinical-semantic variables. Training-derived thresholds were locked before external validation to support both rule-out and rule-in decisions, improve the PPV of high-risk classifications, and facilitate safe de-escalation for low-risk GGNs. We further used SHapley Additive exPlanations (SHAP) [18] to provide transparent patient-level explanations.

## Materials and methods

### Study design and population

This multicentre retrospective cohort study across three institutions (2017–2025) was approved by the ethics committee of the lead institution and complied with the Declaration of Helsinki; informed consent was waived because only de-identified imaging and pathology data were used. Electronic medical records and CT archives (January 2017–July 2025) were searched for patients with GGNs that were resected with histological confirmation or managed as solitary pure ground-glass nodules (pGGNs) under CT surveillance.

**Fig. 1:**
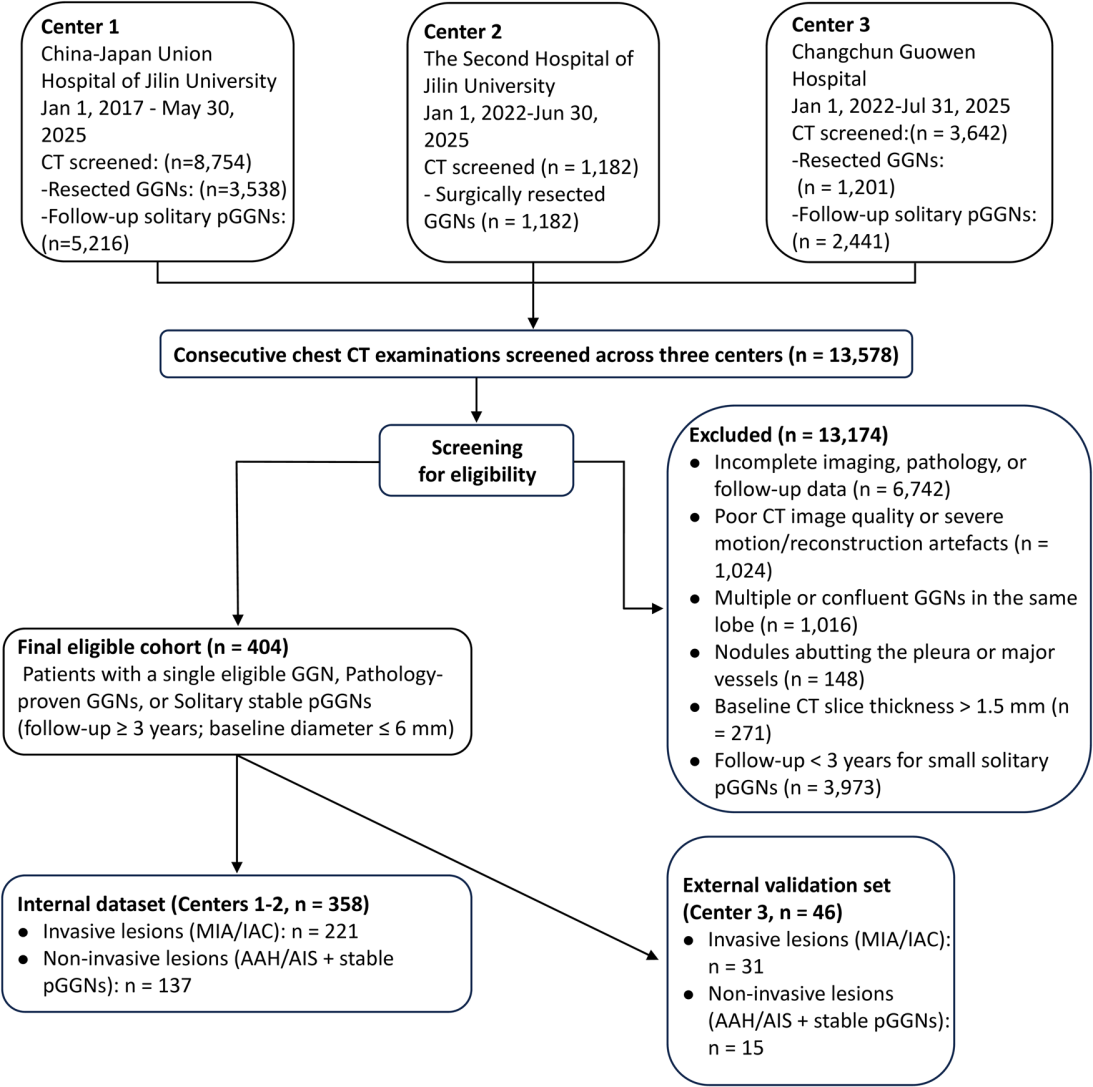
Patient selection and dataset allocation. Chest CT examinations from three centres were screened to identify patients with pulmonary GGNs. Counts excluded at the screening stage are shown according to the corresponding exclusion criteria. After applying the eligibility criteria, 404 patients with 404 GGNs were included. The final cohort was allocated by centre into an internal dataset (Centres 1–2; *n* = 358, including 221 invasive and 137 non-invasive lesions) and an external validation set (Centre 3; *n* = 46, including 31 invasive and 15 non-invasive lesions). The internal dataset was used for model development and internal out-of-fold evaluation

Inclusion criteria were: (1) a GGN confirmed by postoperative pathology, or a solitary pGGN with baseline maximum diameter ≤ 6 mm and radiological stability ≥ 3 years; (2) baseline and follow-up thin-section CT (slice thickness ≤ 1.5 mm); and (3) at least one follow-up CT suitable for quantitative analysis. Exclusions were poor CT quality (e.g. motion or reconstruction artefacts), incomplete imaging, pathology or follow-up data, or unreliable segmentation due to multiple or confluent nodules or close adherence to pleura or large vessels. The patient selection flowchart is shown in Fig. 1.

### Outcome definition and grouping

Pathology was classified according to the 2021 World Health Organization (WHO) classification of thoracic tumours [3]. AAH and AIS were grouped as non-invasive precursor lesions, and MIA and IAC as invasive adenocarcinomas [3, 4]. The primary endpoint was invasive (MIA/IAC) vs non-invasive disease.

To reflect real-world screening practice, the non-invasive group also included a radiologically stable surveillance subgroup, defined as solitary pGGNs with a baseline maximum diameter ≤ 6 mm, at least 3 years of CT follow-up without radiological signs of malignancy, and an annualised absolute relative volume-change rate ≤ 6.0%/year. Derivation of the 6.0%/year volumetric stability threshold from longitudinal volumetric variability in small morphologically stable pGGNs in this cohort is described in the Supplementary Material (Supplementary Figs. S1–S4) and is consistent with prior volumetric variability work [19]. Radiological stability does not fully exclude very slow-growing invasive disease; therefore, we additionally performed a pathology-only sensitivity analysis and evaluated alternative volumetric stability thresholds (see Supplementary Material).

### CT acquisition, measurement and segmentation

CT examinations followed Fleischner Society technical recommendations [6]. Maximum and perpendicular short-axis diameters were measured on axial lung-window images. All measurements were performed on thin-section CT (slice thickness ≤ 1.5 mm) using lung windows (width 1500–1600 Hounsfield units (HU), level −600 to −700 HU). Scanner models and reconstruction settings are summarised in Supplementary Table S1.

Semi-automated volumetry was performed with manual refinement to exclude adjacent vessels, bronchi and chest wall structures, consistent with prior volumetric reproducibility studies [19]. T1 was defined as the CT closest to surgery for resected cases and the last eligible follow-up scan for surveillance cases. Three-dimensional regions of interest (ROIs) were segmented using ITK-SNAP [20], and nodule volume was calculated as voxel count within the ROI multiplied by voxel volume. Inter-observer agreement was assessed in a double-read subset of 30 nodules for volumetry, mean attenuation, and long- and short-axis diameter measurements at baseline and follow-up. Agreement was excellent across all assessed metrics (ICC range, 0.967–0.999; Supplementary Table S2).

### Image harmonisation and growth normalisation

To reduce inter-centre variability, we applied a two-step harmonisation pipeline similar to prior multi-site imaging and radiomics work [21, 22]: slice-sensitivity-profile standardisation followed by resampling to 1.0 × 1.0 × 1.0 mm³ isotropic voxels. Technical settings are provided in the Supplementary Material. To account for variable follow-up intervals, we calculated daily absolute relative volume-change rates from baseline and follow-up volumes normalised by follow-up duration; the exact formulas are provided in the Supplementary Material.

### Radiomic, clinical and derived features

Radiomic features were extracted using PyRadiomics (v3.7.6) from original CT images and Laplacian-of-Gaussian- and wavelet-filtered images, following Image Biomarker Standardisation Initiative (IBSI) guidelines [22, 23]. We computed shape features (from original images only), first-order statistics and texture features, including grey-level co-occurrence matrix (GLCM), grey-level run-length matrix (GLRLM), grey-level size-zone matrix (GLSZM) and grey-level dependence matrix (GLDM) features. Intensities were discretised using a fixed 25-HU bin width without further normalisation to preserve the physical meaning of Hounsfield units [22]. Delta radiomic features for each radiomic variable were defined as daily rates of change between baseline and follow-up values; the exact formula is provided in the Supplementary Material [12–14]. Clinical and semantic variables included age, sex, smoking history, spiculation, pleural traction, vascular convergence and the presence and evolution of a solid component [6, 7]. From the segmentations, we derived baseline and follow-up nodule volume, equivalent spherical diameter (ESD) and mean CT attenuation, plus growth and morphology metrics and selected clinically motivated interaction terms. Rate-type features underwent a log(1 + *x*) transformation. Radiomics study design, feature extraction, modelling and reporting followed the CLEAR (Checklist for Evaluation of Radiomics research) guideline [24].

### Model development, calibration and fusion

**Fig. 2:**
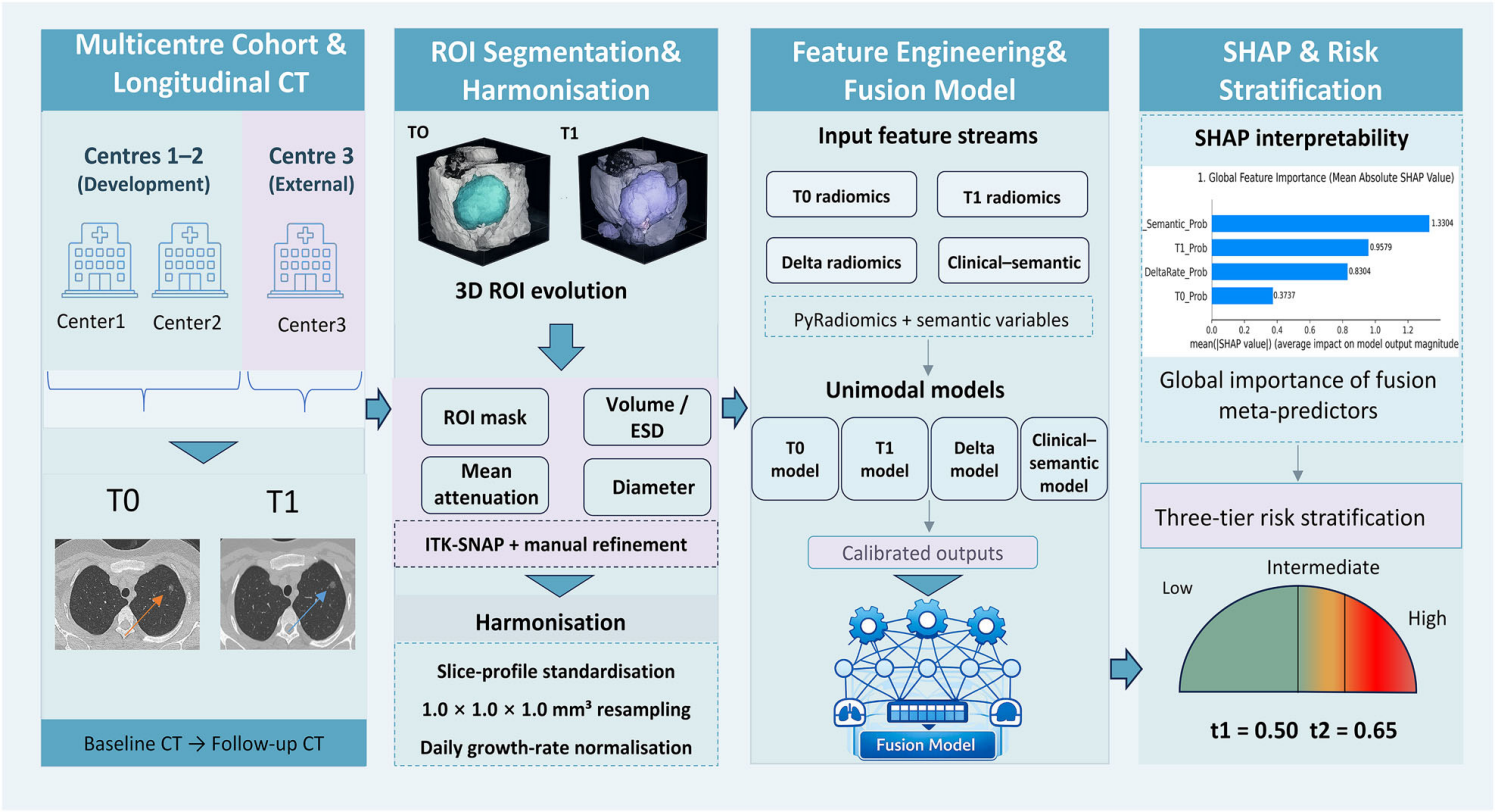
Workflow of the interpretable multi-time-point fusion model for GGN risk stratification. Baseline (T0) and follow-up (T1) thin-section CT scans from three centres were used for three-dimensional ROI segmentation, quantitative measurement, and harmonisation. Radiomic features derived from baseline, follow-up, and longitudinal change were combined with clinical-semantic variables to generate four input feature streams: T0 radiomics, T1 radiomics, delta-radiomics, and clinical-semantic features. Four unimodal models were developed, their out-of-fold predicted probabilities were calibrated, and the calibrated outputs were integrated into a stacked logistic-regression fusion model. The fusion model was then interpreted using SHAP and mapped to three-tier risk stratification using the training-derived and locked thresholds ${t}_{1}=0.50$ and ${t}_{2}=0.65$

We developed separate T0-, T1- and delta-radiomics models, a clinical-semantic model, and a second-layer fusion model. For radiomics, preprocessing, ComBat harmonisation and feature selection were performed strictly within centre-grouped nested cross-validation to prevent information leakage [21]. ComBat parameters were estimated only from the training partition of each fold and applied to the corresponding validation partition; the external cohort was excluded from parameter estimation, and no extrapolative ComBat adjustment was applied to unseen external batches. The final T0-, T1- and delta-radiomics models retained 7, 9 and 42 features, respectively, and the clinical-semantic model retained 6 variables (Supplementary Tables S4 and S5). Calibrated first-layer outputs [25] were combined in a stacked logistic-regression fusion model [26], and internal discrimination was summarised using pooled out-of-fold predictions. Further methodological details are provided in the Supplementary Material. The overall modelling workflow is summarised in Fig. 2.

### Risk stratification and statistical analysis

Fusion-model probabilities were linearly mapped to a 0–100 risk score. Using training out-of-fold predictions, the rule-out threshold $\left({t}_{1}\right)$ was defined as the highest threshold yielding sensitivity ≥ 95% for invasive lesions. The rule-in threshold $\left({t}_{2}\right)$ was first evaluated against PPV ≥ 75%; to preserve a conservative rule-in boundary and a meaningful intermediate-risk band, we additionally required ${t}_{2}\ge {t}_{1}+0.05$ and ${t}_{2}\ge 0.65$. Both thresholds were then locked and applied unchanged to the external cohort.

Discrimination was evaluated using AUC with 95% confidence intervals; calibration using the Brier score, ECE and calibration plots [15]; and clinical utility using decision-curve analysis (DCA) against treat-all, treat-none and guideline-based strategies [16]. Parametric or non-parametric tests were used as appropriate. In the radiomics feature-selection pipeline, univariate screening used Mann–Whitney *U*-tests with Benjamini–Hochberg (BH) false discovery rate correction, retaining features with adjusted *p* < 0.05. AUCs were compared using DeLong’s test [27], and paired proportions using McNemar’s test. Full statistical methods are reported in the Supplementary Material.

### SHAP interpretability, sensitivity analyses and guideline benchmarking

We used SHAP [18] to generate global and case-level explanations for the fusion model. A small web-based prototype for patient-level risk prediction and SHAP visualisation is described in the Supplementary Material (Supplementary Fig. S10) [28]. Two sensitivity analyses were performed: a pathology-only subset and bootstrapped alternative stability thresholds. Finally, we benchmarked the fusion model against Lung-RADS v2022, the 2017 Fleischner guidelines and a recalibrated Brock-lite model [6, 8, 29]. Guideline categories were mapped to three risk tiers using published two-dimensional diameter and growth criteria [6, 8]. Brock-lite was recalibrated using cross-validated Platt scaling [25, 29], and comparisons used AUC, DCA and net reclassification improvement (NRI) [30]. Further methodological details are provided in the Supplementary Material.

## Results

### Patient characteristics

The final cohort comprised 404 patients with GGNs, including 358 in the training cohort and 46 in the external cohort (31 invasive, 15 non-invasive; Fig. 1). Because external validation was centre-based, Centres 1–2 were used internally and Centre 3 served as an independent external cohort; the size imbalance also reflects the smaller number of Centre-3 cases meeting all eligibility criteria, especially the requirement for ≥ 3 years of CT follow-up in the radiologically stable surveillance subgroup. In the training cohort, 221 lesions were invasive and 137 non-invasive. Invasive lesions occurred more often in older and female patients and showed larger baseline volume, higher mean CT attenuation and more malignant semantic features than non-invasive lesions (all *p* < 0.05; Tables 1 and 2). Similar patterns were observed in the external cohort.

**Table 1:** Baseline characteristics of patients with invasive and non-invasive GGNs in the training and validation cohorts

	Training cohort				Validation cohort			
Variable	Overall (*n* = 358)	Invasive GGN (*n* = 221)	Non-invasive GGN (*n* = 137)	*p* value^1^	Overall (*n* = 46)	Invasive GGN (*n* = 31)	Non-invasive GGN (*n* = 15)	*p* value^2^
Age (years)	57.00 [49.00, 64.00]	59.00 [51.00, 65.00]	55.00 [44.00, 62.00]	**< 0.001**	52.54 ± 12.52	52.10 ± 13.17	53.47 ± 11.44	0.72
Sex, *n* (%)				**0.017**				> 0.99
Male	104 (29%)	54 (24%)	50 (36%)		8 (17%)	5 (16%)	3 (20%)	
Female	254 (71%)	167 (76%)	87 (64%)		38 (83%)	26 (84%)	12 (80%)	
Smoker (yes), *n* (%)	49 (14%)	30 (14%)	19 (14%)	> 0.99	4 (8.7%)	4 (13%)	0 (0%)	0.29
Spiculation (yes), *n* (%)	99 (28%)	87 (39%)	12 (8.8%)	**< 0.001**	14 (30%)	14 (45%)	0 (0%)	**0.002**
Lobulation (yes), *n* (%)	146 (41%)	103 (47%)	43 (31%)	**0.006**	22 (48%)	19 (61%)	3 (20%)	**0.012**
Vacuole (yes), *n* (%)	108 (30%)	88 (40%)	20 (15%)	**< 0.001**	14 (30%)	13 (42%)	1 (6.7%)	**0.018**
Air bronchogram (yes), *n* (%)	144 (40%)	116 (52%)	28 (20%)	**< 0.001**	20 (43%)	20 (65%)	0 (0%)	**< 0.001**
Vascular convergence (yes), *n* (%)	227 (63%)	186 (84%)	41 (30%)	**< 0.001**	30 (65%)	28 (90%)	2 (13%)	**< 0.001**
Pleural indentation (yes), *n* (%)	78 (22%)	64 (29%)	14 (10%)	**< 0.001**	11 (24%)	10 (32%)	1 (6.7%)	0.074
Initial solid component (yes), *n* (%)	74 (21%)	71 (32%)	3 (2.2%)	**< 0.001**	20 (43%)	19 (61%)	1 (6.7%)	**< 0.001**
Long diameter (mm), median [Q1, Q3]	8.00 [5.00, 10.00]	9.00 [8.00, 12.00]	5.00 [4.00, 6.00]	**< 0.001**	9.00 [6.00, 12.00]	11.00 [9.00, 14.00]	5.00 [4.00, 6.00]	**< 0.001**
Short diameter (mm), median [Q1, Q3]	6.00 [4.00, 8.00]	7.00 [6.00, 9.00]	4.00 [3.00, 5.00]	**< 0.001**	7.00 [5.00, 9.00]	8.00 [7.00, 10.00]	5.00 [4.00, 5.00]	**< 0.001**
Baseline volume (mL), median [Q1, Q3]	0.26 [0.09, 0.54]	0.40 [0.24, 0.79]	0.07 [0.03, 0.15]	**< 0.001**	0.39 [0.15, 0.99]	0.69 [0.38, 1.11]	0.11 [0.04, 0.17]	**< 0.001**
Baseline volume (mm³), median [Q1, Q3]	255.31 [93.03, 540.06]	399.41 [235.92, 785.00]	73.18 [32.80, 147.67]	**< 0.001**	386.30 [146.74, 994.36]	692.08 [384.12, 1,114.47]	110.76 [36.41, 169.84]	**< 0.001**
Baseline mean CT (HU), median [Q1, Q3]	−660.90 [−738.58, −563.66]	−631.66 [−709.14, −531.37]	−706.27 [−763.66, −626.31]	**< 0.001**	−575.75 [−664.05, −528.48]	−552.13 [−613.07, −513.21]	−645.34 [−729.76, −593.00]	**< 0.001**
Location, *n* (%)				0.053				0.91
Right upper lobe	150 (42%)	94 (43%)	56 (41%)		21 (46%)	13 (42%)	8 (53%)	
Right middle lobe	21 (5.9%)	13 (5.9%)	8 (5.8%)		3 (6.5%)	2 (6.5%)	1 (6.7%)	
Right lower lobe	59 (16%)	43 (19%)	16 (12%)		7 (15%)	6 (19%)	1 (6.7%)	
Left upper lobe	88 (25%)	54 (24%)	34 (25%)		12 (26%)	8 (26%)	4 (27%)	
Left lower lobe	40 (11%)	17 (7.7%)	23 (17%)		3 (6.5%)	2 (6.5%)	1 (6.7%)	
Nodule shape, *n* (%)				0.66				**< 0.001**
Regular	164 (46%)	99 (45%)	65 (47%)		19 (41%)	7 (23%)	12 (80%)	
Irregular	194 (54%)	122 (55%)	72 (53%)		27 (59%)	24 (77%)	3 (20%)	

Data are presented as mean ± standard deviation or median [interquartile range] for continuous variables and as *n* (% of patients) for categorical variables, unless otherwise indicated. Comparisons between invasive and non-invasive GGNs were performed using Welch’s *t*-test for approximately normally distributed continuous variables, the Wilcoxon rank-sum test for non-normally distributed continuous variables, and the Chi-square test or Fisher’s exact test (with Monte Carlo simulation where appropriate) for categorical variables. *p* values are two-sided; bold *p* values indicate statistical significance at α = 0.05

Baseline characteristics of patients with invasive and non-invasive GGNs in the training and validation cohorts

*GGN* ground-glass nodule, *SD* standard deviation, *IQR* interquartile range, *HU* Hounsfield unit

**Table 2:** Follow-up and delta CT characteristics of invasive and non-invasive GGNs in the training and validation cohorts

	Training cohort				Validation cohort			
Variable	Overall (*n* = 358)	Invasive GGN (*n* = 221)	Non-invasive GGN (*n* = 137)	*p* value^1^	Overall (*n* = 46)	Invasive GGN (*n* = 31)	Non-invasive GGN (*n* = 15)	*p* value^2^
Follow-up days, Median [Q1, Q3]	698.00 [234.00, 1491.00]	413.00 [172.00, 892.00]	1517.00 [581.00, 1761.00]	**< 0.001**	205.00 [39.00, 1097.00]	71.00 [31.00, 368.00]	1107.00 [1097.00, 1119.00]	**< 0.001**
Follow-up volume (mL), Median [Q1, Q3]	0.35 [0.11, 0.79]	0.60 [0.36, 1.14]	0.07 [0.03, 0.19]	**< 0.001**	0.46 [0.15, 0.98]	0.72 [0.44, 1.59]	0.12 [0.03, 0.15]	**< 0.001**
Follow-up mean CT (HU), Median [Q1, Q3]	−648.19 [−733.36, −556.93]	−622.53 [−711.96, −538.53]	−674.56 [−750.97, −614.44]	**< 0.001**	−594.08 [−637.88, −533.98]	−560.17 [−626.94, −509.97]	−637.49 [−703.28, −587.91]	**0.003**
Long diameter at T1 (mm), Median [Q1, Q3]	9.00 [5.00, 12.00]	11.00 [9.00, 13.00]	5.00 [4.00, 7.00]	**< 0.001**	9.50 [6.00, 13.00]	11.00 [9.00, 15.00]	5.00 [4.00, 6.00]	**< 0.001**
Short diameter at T1 (mm), Median [Q1, Q3]	7.00 [4.00, 9.00]	8.00 [7.00, 11.00]	4.00 [3.00, 5.00]	**<0.001**	7.00 [5.00, 9.00]	9.00 [7.00, 11.00]	5.00 [4.00, 5.00]	**<0.001**
Volume doubling time (days), Median [Q1, Q3]	856.35 [154.26, 3512.44]	770.55 [309.45, 1621.34]	1617.22 [−4068.28, 8899.18]	0.14	202.24 [−2058.85, 1262.70]	310.10 [47.27, 1256.10]	−3430.87 [−13,140.19, 4769.63]	0.11
ΔMean HU per day, Median [Q1, Q3]	0.00 [−0.04, 0.06]	0.00 [−0.07, 0.07]	0.00 [−0.02, 0.04]	0.44	−0.01 [−0.07, 0.13]	−0.03 [−0.38, 0.29]	0.00 [−0.01, 0.04]	0.43
ΔCompactness proxy, Median (IQR)	−0.01 (−0.53 – 0.21)	−0.07 (−0.73 – 0.19)	0.08 (−0.19 – 0.23)	> 0.99	0.00 (−0.02 – 0.14)	0.09 (−0.11 – 0.20)	0.00 (0.00 – 0.00)	> 0.99
Solid component increase (Yes), *n* (%)	73 (20%)	70 (32%)	3 (2.2%)	**< 0.001**	4 (8.7%)	4 (13%)	0 (0%)	0.29

Follow-up and delta metrics are calculated in patients with at least two CT examinations; missing values, therefore, reflect absent follow-up. Data are presented as mean ± standard deviation or median [interquartile range] for continuous variables and as *n* (% of patients) for categorical variables, unless otherwise stated. Comparisons between invasive and non-invasive GGNs were performed using Welch’s *t*-test, Wilcoxon rank-sum test, the Chi-square test or Fisher’s exact test (with simulated *p* values where appropriate). *p* values are two-sided; bold *p* values indicate statistical significance at α = 0.05

Follow-up and delta characteristics of invasive and non-invasive GGNs in the training and validation cohorts

*GGN* ground-glass nodule, *SD* standard deviation, *IQR* interquartile range, *HU* Hounsfield unit, *T1* follow-up time point

### Unimodal model performance

**Fig. 3:**
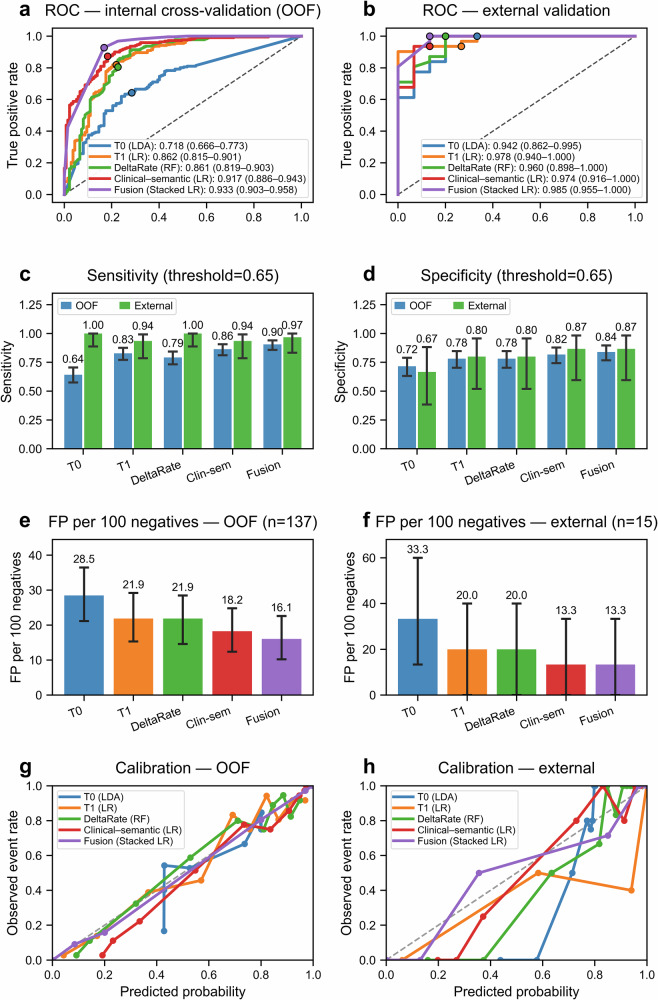
Discrimination, false-positive burden, and calibration of the unimodal and fusion models at the training-derived and locked thresholds (${t}_{1}=0.50$, ${t}_{2}=0.65$). **a**, **b** Show receiver operating characteristic (ROC) curves for the four unimodal models (T0 radiomics, T1 radiomics, delta-radiomics, and clinical-semantic) and the stacked fusion model in internal cross-validation (out-of-fold predictions, **a**) and the external validation cohort (**b**). **c**, **d** Display sensitivity and specificity, respectively, with 95% confidence intervals at the rule-in threshold ${t}_{2}=0.65$ for each model in internal cross-validation (out-of-fold predictions) and the external cohort. **e**, **f** Show the number of false-positive high-risk classifications per 100 non-invasive lesions (false positives per 100 negatives) for the different models in the internal (**e**) and external (**f**) cohorts at ${t}_{2}=0.65$. **g**, **h** Depict calibration curves in internal cross-validation (**g**) and external validation (**h**), using decile-based bins to compare predicted probabilities with observed event rates; the dashed line indicates perfect calibration. For the externally calibrated fusion model, the exact calibration metrics were ECE = 0.060 and MCE = 0.500. Overall, the fusion model showed strong discrimination, a lower false-positive burden, and favourable calibration compared with the unimodal models

In centre-grouped cross-validation, AUCs were 0.718 for T0-radiomics, 0.862 for T1-radiomics, 0.861 for delta-radiomics, and 0.917 for the clinical-semantic model (Fig. 3a and Supplementary Table S6A). External AUCs remained high for all unimodal models (0.942–0.978; Fig. 3b and Supplementary Table S6A). Full 95% confidence intervals are provided in Supplementary Table S6A. At ${t}_{2}=0.65$, false-positive high-risk classifications per 100 non-invasive lesions in the external cohort were 33.3, 20.0, 20.0, and 13.3, respectively (Fig. 3f).

### Stacked fusion model and risk stratification

The calibrated stacked fusion model, combining all unimodal outputs, showed the best overall performance. Its AUC was 0.933 (95% CI: 0.903–0.958) in centre-grouped cross-validation and 0.985 (95% CI: 0.955–1.000) in the external cohort, exceeding that of all unimodal models (Fig. 3a, b and Supplementary Table S6A). Calibration in the external cohort was good (Brier score 0.042; expected calibration error 0.060; Fig. 3g, h).

**Fig. 4:**
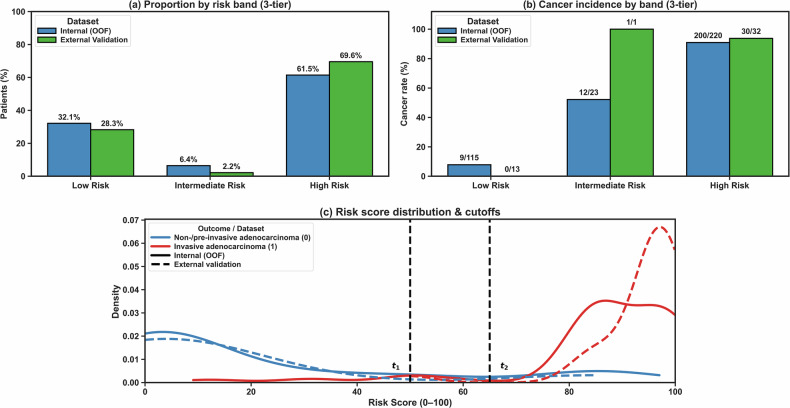
Three-tier risk stratification using the calibrated fusion model. Predicted probabilities were mapped to three risk bands using the training-derived and locked thresholds ${t}_{1}=0.50$ (low-risk rule-out) and ${t}_{2}=0.65$ (high-risk rule-in). **a** Shows the proportion of patients assigned to the low-, intermediate-, and high-risk bands in internal out-of-fold (OOF) evaluation and external validation. **b** Shows cancer incidence within each risk band in internal OOF evaluation and external validation. **c** Shows the distribution of fusion-model risk scores (0–100) for non-invasive and invasive lesions in internal OOF evaluation and external validation; the vertical dashed lines indicate ${t}_{1}$ and ${t}_{2}$, corresponding to risk scores of 50 and 65. Most invasive lesions were concentrated in the high-risk band, whereas event rates remained very low in the low-risk band

Using the training-derived and locked thresholds ${t}_{1}=0.50$ and ${t}_{2}=0.65$, predicted probabilities were mapped to three risk bands. In the training cohort, 31.6% of patients were classified as low risk, 6.4% as intermediate risk and 62.0% as high risk, with corresponding cancer rates of 6.2%, 60.9% and 90.1%, respectively (Fig. 4a, b). In the external cohort, 28.3% of patients (13/46) were low risk, 2.2% (1/46) intermediate risk and 69.6% (32/46) high risk, and cancer incidence by band was 0%, 100% and 93.8%, respectively. This yielded a negative predictive value (NPV) of 100.0% (95% CI: 75.3–100.0) for low-risk classifications and a PPV of 93.8% (95% CI: 79.2–99.2) for high-risk classifications (Supplementary Table S6B).

Risk-score distributions showed clear separation between invasive and non-invasive lesions (Fig. 4c). At the high-risk threshold ${t}_{2}=0.65$, the fusion model reduced false-positive high-risk classifications in the external cohort from 33.3 to 13.3 per 100 non-invasive lesions compared with the T0-radiomics model, while maintaining high sensitivity (96.8%; 30/31 invasive lesions correctly classified as high risk; Fig. 3f and Supplementary Table S6B). Similar reductions were seen in internal cross-validation (Fig. 3e).

### Benchmark comparisons

**Fig. 5:**
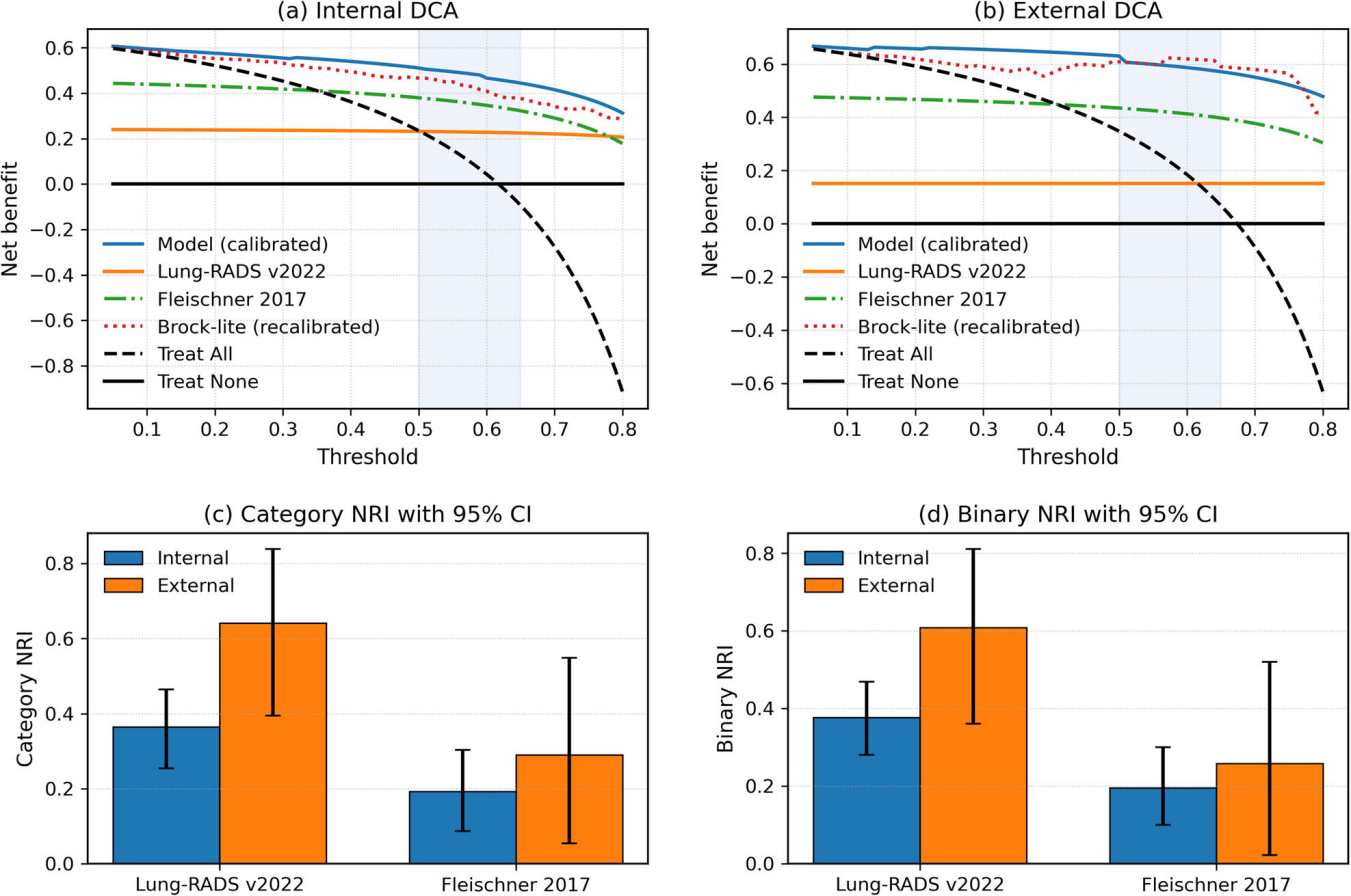
DCA and NRI for the calibrated fusion model compared with guideline-based and clinical risk models. **a**, **b** Show internal (out-of-fold) and external DCAs, respectively, comparing the net benefit of the fusion model (blue solid line) with Lung-RADS v2022, the 2017 Fleischner guidelines, a recalibrated Brock-lite model, and treat-all and treat-none strategies across a range of threshold probabilities. The shaded region indicates the interval around the training-derived and locked rule-out and rule-in thresholds (${t}_{1}=0.50$, ${t}_{2}=0.65$). **c**, **d** Display categorical NRI and binary NRI, respectively, with 95% confidence intervals, for the fusion model vs Lung-RADS v2022 and the 2017 Fleischner guidelines in the internal and external cohorts. Positive NRI values indicate improved risk classification with the fusion model compared with the guideline strategies

Compared with Lung-RADS v2022 and the 2017 Fleischner guidelines, the calibrated fusion model showed higher net benefit across threshold probabilities of 0.10–0.80 in both internal and external decision-curve analyses (Fig. 5a, b). Categorical NRI vs Lung-RADS was 0.36 internally, and 0.64 externally; the corresponding values vs the Fleischner guidelines were 0.19 and 0.29 (Fig. 5c and Supplementary Table S6C). Gains were driven mainly by correct upward reclassification of malignant lesions, with limited worsening among non-invasive cases (Fig. 5d and Supplementary Table S6C).

### SHAP interpretability and sensitivity analyses

**Fig. 6:**
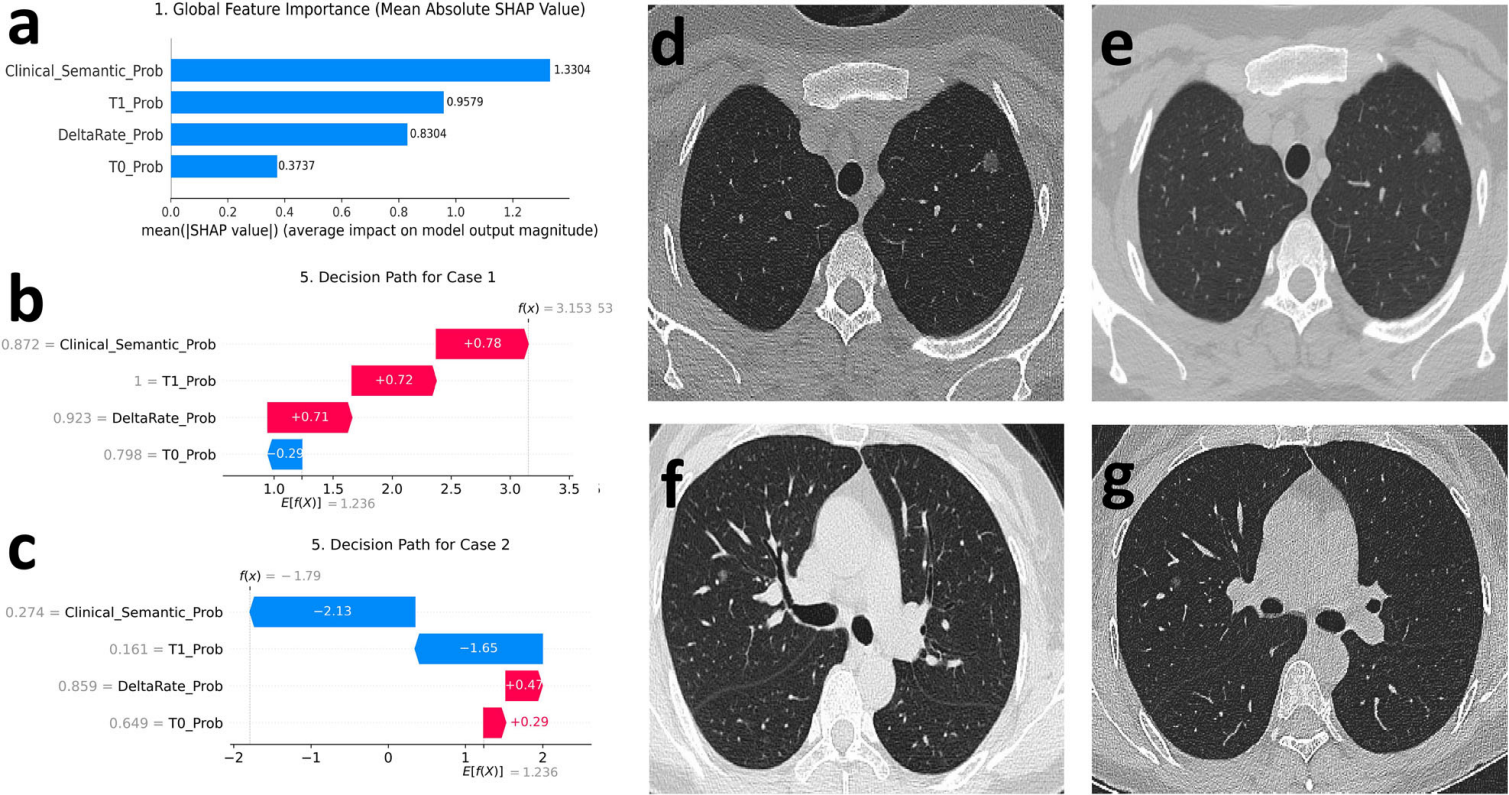
SHAP-based interpretability and illustrative cases for the calibrated fusion model. **a** Global mean absolute SHAP values for the four meta-predictors (T0 radiomics, T1 radiomics, delta-radiomics and the clinical–semantic model), showing that the clinical–semantic probability and T1/delta components contribute most to the final risk score, whereas the T0-only component has a smaller impact. **b** SHAP decision path for a representative invasive lesion correctly classified as high risk (prediction above the rule-in threshold ${t}_{2}$), with additive positive contributions from the clinical–semantic, T1 and delta-radiomics meta-predictors and a small negative contribution from the T0 component. **c** SHAP decision path for a representative non-invasive lesion classified as low risk (prediction below the rule-out threshold ${t}_{1}$), where negative contributions from the clinical–semantic and T1 components dominate and outweigh small positive contributions from the radiomics-only components. **d**, **e** Baseline (T0) and follow-up (T1) thin-section CT images of the high-risk invasive lesion in (**b**), demonstrating an enlarging subsolid nodule with interval increase in attenuation. **f**, **g** Baseline and follow-up CT images of the low-risk non-invasive lesion in panel c, showing a small pure GGN with radiographic stability over time

Global SHAP analysis showed that the clinical-semantic meta-predictor contributed most to fused decisions, followed by T1- and delta-radiomics, whereas T0-radiomics contributed less (Fig. 6a), consistent with unimodal performance. Case-level SHAP plots illustrated how the four meta-predictors combined in individual patients (Fig. 6b–g). Fusion ablation showed no material AUC change after removing T0 radiomics, whereas removing T1 or delta produced only small decreases with confidence intervals crossing zero (Supplementary Fig. S13).

In the pathology-only sensitivity analysis (*n* = 305), baseline and follow-up patterns were broadly similar to those in the full cohort (Supplementary Tables S7 and S8). The fusion model retained high discrimination (external AUC 0.935 vs 0.828 for T0-radiomics; *p* = 0.036) and yielded fewer false-positive high-risk classifications than unimodal radiomics at ${t}_{2}=0.65$ in internal cross-validation (McNemar *p* < 0.01), with similar trends externally (Supplementary Table S9A).

## Discussion

In this multicentre study, we developed and externally validated an interpretable fusion model integrating clinical-semantic variables with baseline, follow-up and delta-radiomics for the prediction of GGN invasiveness. The model showed high discrimination (external AUC 0.985), good calibration, 100% NPV in the low-risk group, and 93.8% PPV in the high-risk group, while assigning only 2.2% of patients to an intermediate-risk grey zone.

These findings are clinically relevant for LDCT screening and incidental GGN management. Guideline-based strategies rely mainly on two-dimensional size and simple growth criteria, which provide limited specificity and lead to false-positive classifications and prolonged surveillance of many indolent lesions, whereas some apparently stable GGNs later prove invasive [1, 2, 6–9]. By incorporating temporal radiomics and calibrated probabilities, our model reduced false-positive high-risk classifications compared with the T0-radiomics model while maintaining high sensitivity and NPV at the locked rule-out threshold, suggesting a more favourable balance between early detection and overtreatment.

Methodologically, this study extends prior radiomics and delta-radiomics work [12–14]. Rather than pooling all variables into a single high-dimensional feature block, we trained separate baseline, follow-up and delta-radiomics models and combined them with a clinical-semantic model through calibrated stacking. The stronger performance of T1- and delta-radiomics relative to T0-radiomics, together with the dominant contribution of the clinical-semantic component, suggests that temporal evolution adds prognostic information beyond static morphology while interpretable semantic features remain a robust anchor for decision-making [6, 7, 12, 31]. We also evaluated calibration, decision-curve performance and false-positive burden, in line with contemporary recommendations for radiomics and prediction-model reporting [15, 16, 22, 24].

The study population and reference standard are additional strengths. Many radiomics studies are based on surgical series, which over-represent aggressive lesions and under-represent small GGNs managed conservatively [13, 14, 31]. Our composite reference standard combined WHO-defined AAH/AIS with a surveillance subgroup of small solitary pGGNs that remained radiologically stable for at least three years with constrained annualised volumetric growth [3–5, 19], thereby better reflecting outpatient and screening populations. The pathology-only sensitivity analysis showed that the fusion model retained high discrimination and a lower false-positive burden than the unimodal radiomics models, partly mitigating concerns about verification bias.

Compared with Lung-RADS, the Fleischner guidelines and recalibrated Brock-lite, the fusion model showed greater net benefit across clinically relevant thresholds and favourable reclassification, driven mainly by correct upward reclassification of malignant lesions [6, 8, 29, 30]. These findings suggest that combining three-dimensional volumetry, longitudinal radiomics and semantic information offers incremental prognostic value beyond diameter-based schemes and established risk models.

SHAP analysis helped explain model behaviour. The limited incremental contribution of T0 radiomics likely reflects overlap with baseline semantic and size-related descriptors, whereas follow-up and longitudinal components capture more informative temporal evolution. At the meta-predictor level, the clinical-semantic component contributed most, followed by T1- and delta-radiomics. Within that branch, nodule size, solid components, spiculation and vascular convergence were most influential, consistent with prior literature [6, 7, 17, 32]. Case-level SHAP plots showed that concordant evidence across modalities supported high-confidence rule-in decisions, whereas discordant patterns were associated with lower final risk.

This study has limitations. It was retrospective and, although multicentre, the external validation cohort was small and imbalanced, especially for non-invasive lesions; accordingly, calibration summaries and threshold-dependent estimates should be interpreted cautiously because several 95% confidence intervals were wide, and larger prospective multicentre studies are needed to confirm threshold reliability and generalisability. Residual heterogeneity in CT acquisition and reconstruction may persist despite slice-profile standardisation and ComBat harmonisation [21, 22], particularly for texture features that are reconstruction dependent, and reproducibility beyond the three participating centres remains uncertain [33, 34]. Only two time points were modelled; denser longitudinal sampling may better capture non-linear growth [13, 14]. The composite reference standard cannot eliminate verification bias: radiological stability does not guarantee benignity, very slow-growing invasive lesions may have been misclassified, and the 6.0%/year stability threshold was derived internally and may vary across scanners or follow-up protocols. Nevertheless, the pathology-only and alternative-threshold sensitivity analyses showed consistent trends. Finally, the web-based SHAP tool is intended for research transparency rather than routine clinical deployment and should not inform patient care without prospective validation and real-world decision-impact analysis or simulation.

## Conclusions

We developed and externally validated an interpretable fusion model combining clinical-semantic features with baseline, follow-up and delta-radiomics for prediction of GGN invasiveness. The model showed high discrimination and good calibration, supported three-tier risk stratification with strong rule-out and rule-in performance, and reduced false-positive high-risk classifications vs unimodal radiomics and guideline-based strategies. These findings support prospective evaluation of calibrated, interpretable fusion models for evolution-based GGN management and reduction of overtreatment in LDCT screening.

## Supplementary information

ELECTRONIC SUPPLEMENTARY MATERIAL

## Data Availability

The de-identified imaging and clinical datasets generated and analysed during the current study are not publicly available owing to institutional and ethical restrictions, but may be made available from the corresponding author on reasonable request and with permission from the participating centres and ethics committee.

## Abbreviations

AAH: Atypical adenomatous hyperplasia
AIS: Adenocarcinoma in situ
AUC: Area under the receiver operating characteristic curve
DCA: Decision-curve analysis
GGN: Ground-glass nodule
IAC: Invasive adenocarcinoma
LDCT: Low-dose computed tomography
Lung-RADS: Lung CT screening reporting and data system
MIA: Minimally invasive adenocarcinoma
NPV: Negative predictive value
NRI: Net reclassification improvement
pGGN: Pure ground-glass nodule
PPV: Positive predictive value
SHAP: SHapley Additive exPlanations
