# Supplementary material for: CT-based interpretable delta-radiomics model for risk stratification of pulmonary ground-glass nodules: a multicentre study
Source: Insights Imaging. 2026 May 14;17:126. doi: 10.1186/s13244-026-02297-2 (PMC13172203; doi:10.1186/s13244-026-02297-2)
Supplement: Supplementary file 1 — ELECTRONIC SUPPLEMENTARY MATERIAL [file 13244_2026_2297_MOESM1_ESM.docx]

# Supplementary Material

**Derivation of the 6.0%/year volumetric stability threshold**

**S1.1 Stable cohort definition.**

To derive a volumetric stability threshold for small pure ground-glass nodules (pGGNs), we defined an internal empirical stability set (“stable cohort”) within the overall 404-patient study cohort. This set consisted of solitary pGGNs fulfilling all of the following criteria: (1) baseline maximum axial diameter ≤ 6 mm; (2) thin-section CT (slice thickness ≤ 1.5 mm) available at baseline and follow-up; (3) continuous radiological surveillance for at least 3 years without development of a solid component, lobulation, spiculation, or other radiological signs of malignancy; (4) no interval growth deemed suspicious by the multidisciplinary team; and (5) no therapeutic intervention directed at the nodule during follow-up. In total, 99 nodules met these criteria.

Note on generalisability and verification bias: Although the stable cohort definition required ≥3 years of CT follow-up, absence of malignant interval change, and constrained annualised volumetric change, radiological stability is not equivalent to pathological confirmation and very slow-growing invasive lesions cannot be completely excluded. In addition, volumetric variability may differ across scanners, reconstruction settings, and longitudinal follow-up protocols. We therefore interpret the 6.0%/year criterion as a pragmatic, high-specificity operating definition within this multicentre dataset and provide sensitivity analyses using alternative thresholds.

**S1.2 Growth-rate definitions.**

For each nodule in the stable cohort, baseline (T0) and follow-up (T1) volumes, denoted $V_{0}$

and $V_{1}$, were obtained from semi-automated segmentations. The follow-up interval was expressed as the number of days between scans ($\mathrm{FollowupDays}$). The daily absolute relative volume-change rate was defined as

$$r_{\text{day}}=\frac{\left| V_{1}-V_{0} \right|}{V_{0}\times\text{FollowupDays}}$$

The corresponding annualised rate was defined as

$$r_{\text{year}}=r_{\text{day}}\times365$$

This formulation captures relative volumetric variability per unit time and is analogous to previously used growth metrics for pulmonary nodules.

**S1.3 Empirical percentile-based threshold.**

The empirical distribution of $r_{\text{year}}$ in the stable cohort was examined. Upper percentiles of this distribution were considered as candidate thresholds for volumetric stability. The 95th percentile was selected to limit the expected proportion of truly stable nodules exceeding the threshold to approximately 5%. The corresponding value was approximately 0.0588/year, which we rounded to a pragmatic 6.0%/year criterion for use in the main analysis. Thus, nodules with an annualised absolute relative volume-change rate of $r_{\mathrm{year}}\leq6.0\%/year$ were classified as radiographically stable for the purpose of defining the non-invasive surveillance subgroup.

**S1.4 Bootstrap and sensitivity analyses.**

To assess sampling variability of the percentile estimates, we performed non-parametric bootstrap resampling of the stable cohort. In each bootstrap sample (nodules resampled with replacement), the empirical distribution of $r_{\mathrm{year}}$ was recomputed, and the 90th, 95th, and 97.5th percentiles were recorded. The resulting bootstrap distributions were used to obtain confidence intervals for these percentiles and to assess the robustness of the 95th percentile as a working stability threshold. Sensitivity analyses examining alternative thresholds (e.g. 4.0%, 5.0%, 7.0%, and 8.0%/year) are reported in the the Supplementary Material.

**S1.5 Sensitivity analyses and internal specificity of the stability threshold**

Using the stable cohort described above, we further examined the distributional properties and internal specificity of the proposed threshold. In this cohort, the median annualised absolute relative volume change was 0.025/year, with the 75th, 90th, 95th, and 97.5th percentiles equal to 0.041/year, 0.054/year, 0.059/year, and 0.066/year, respectively. We therefore used the 95th percentile (~0.059/year) as a working definition of radiographic stability and rounded it to a pragmatic 6.0%/year threshold in the main analysis. In sensitivity analyses, we also examined alternative thresholds close to the 90th and 97.5th percentiles (approximately 5.4%/year and 6.6%/year).

Figure S1 shows a scatter plot of baseline volume ​$V_{0}$ versus follow-up volume $V_{1}$ in the stable cohort, with most points lying close to the identity line $V_{1}=V_{0}$, indicating only small volume changes. Figure S2 presents the empirical cumulative distribution function (ECDF) of the annualised absolute relative volume change $\left| \Delta V \right|/V_{0}$; most nodules had values below 4%/year, and the 90th–97.5th percentiles clustered between 5% and 7%/year. Figure S3 shows the corresponding histogram, confirming that the distribution was concentrated in the low-change range with a relatively short right tail.

To quantify the internal specificity of candidate thresholds, we performed a threshold scan over a grid of values from 0.02 to 0.50/year. For each threshold, we calculated the proportion of nodules in the stable cohort that would be flagged as “growing” (flag rate, i.e. false-positive rate), together with 95% confidence intervals obtained by non-parametric bootstrap resampling (Figure S4). As expected, the flag rate decreased monotonically as the threshold increased. Around 0.054/year (the internal 90th percentile), approximately 10% of stable nodules would have been incorrectly flagged as growing. At 0.059/year (the 95th percentile, corresponding to the chosen 6.0%/year threshold), the flag rate was about 5% (95% CI approximately 1%–10%). At 0.066/year (the 97.5th percentile), the flag rate further decreased to about 3%. For comparison, we also plotted a 25%/year reference line corresponding to a commonly used volume-change threshold in the literature; at this level, virtually no nodule in the stable cohort would have been flagged as growing.

We additionally examined the robustness of percentile estimates across data sources. When the cohort was stratified by two internal data sources, the 95th percentile of the annualised absolute relative volume change was approximately 0.055/year and 0.083/year, respectively, compared with 0.059/year in the pooled cohort. This suggests some heterogeneity between sources but also confirms that the chosen 6.0%/year threshold lies in the upper tail of the internal distribution, providing high specificity and reasonable robustness.

The sensitivity analysis was implemented in Python using pandas and matplotlib. The script, which can be provided as an additional electronic resource if requested, filters the stable cohort, computes the annualised absolute relative volume change and related metrics, estimates percentiles and bootstrap confidence intervals, and generates the ECDF, histogram, baseline–follow-up scatter plot, and threshold–flag-rate curve shown in Figures S1–S4.

**
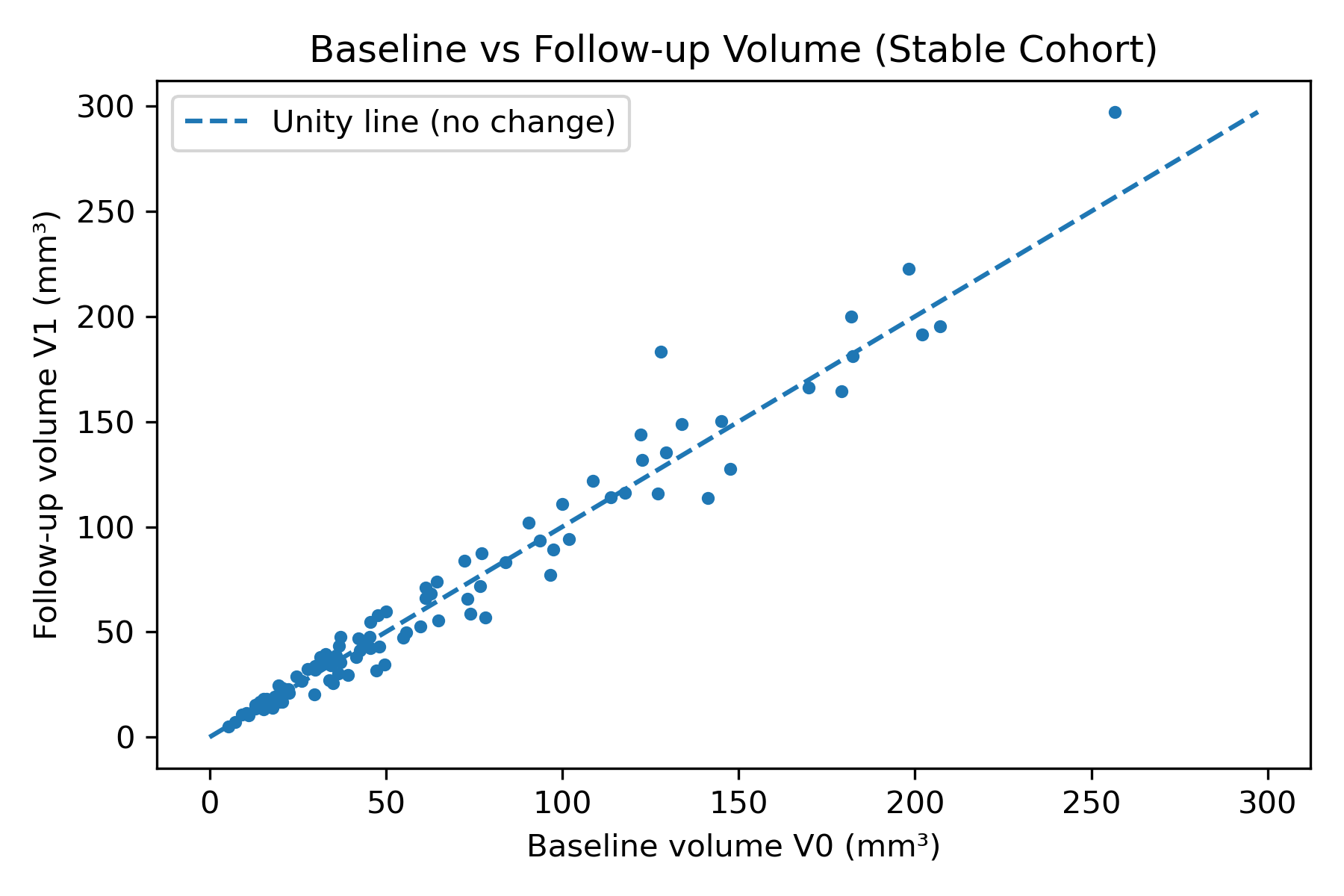
**

**Figure S1. Baseline versus follow-up volume in the stable cohort.**

*Scatter plot of baseline volume* $V_{0}$*against follow-up volume* $V_{1}$*for the internally defined stable pGGN cohort (n = 99). The dashed line indicates the identity line* $V_{1}=V_{0}$*; most nodules cluster around this line, consistent with small volumetric changes over ≥3 years of follow-up.*

**
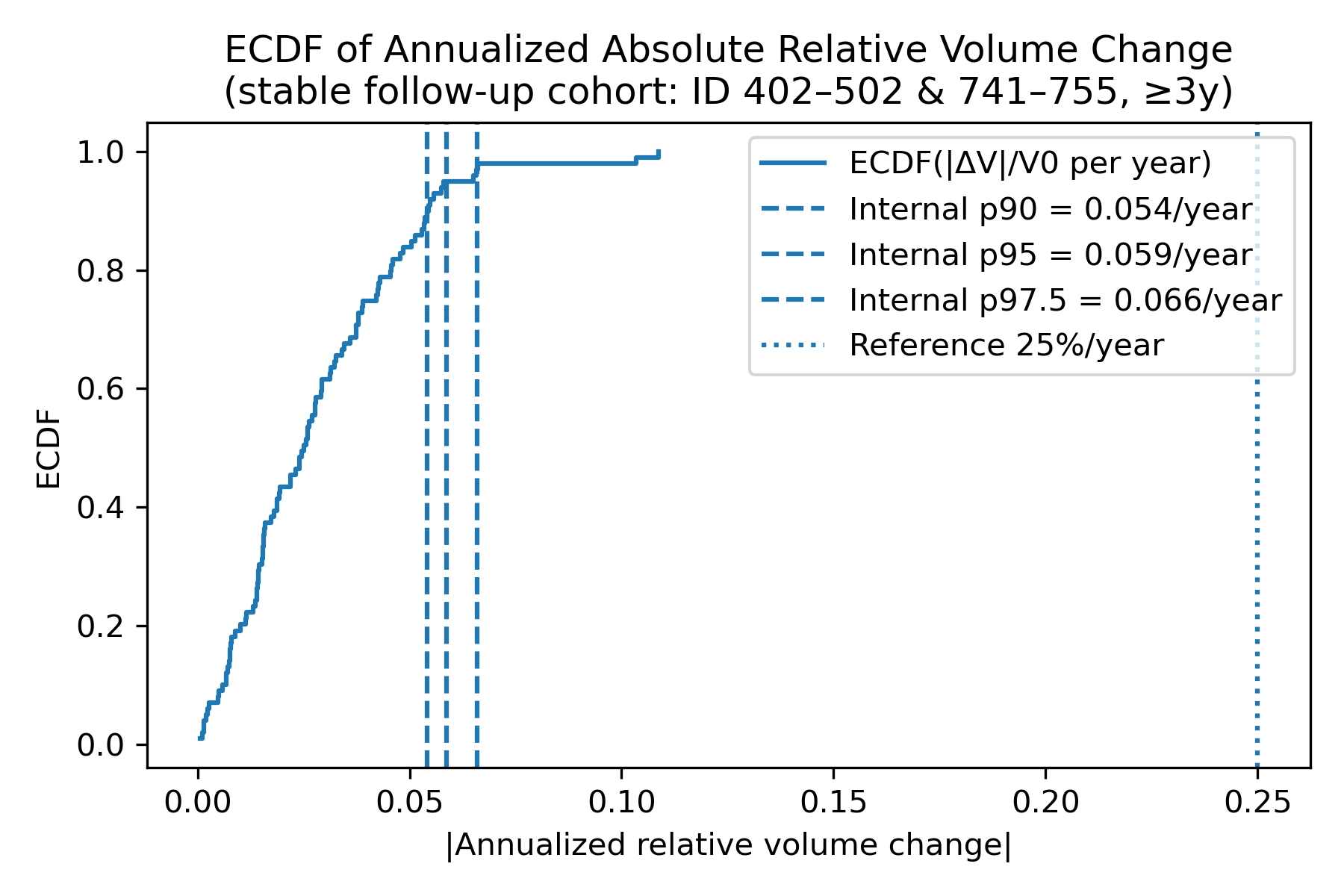
**

**Figure S2. ECDF of annualised absolute relative volume change.**

*Empirical cumulative distribution function of the annualised absolute relative volume change* $\mid\Delta V\mid/V_{0}$*in the stable cohort. Vertical dashed lines indicate the internal 90th, 95th and 97.5th percentiles (approximately 0.054, 0.059 and 0.066/year, respectively); the dotted line marks a 25%/year reference threshold.*


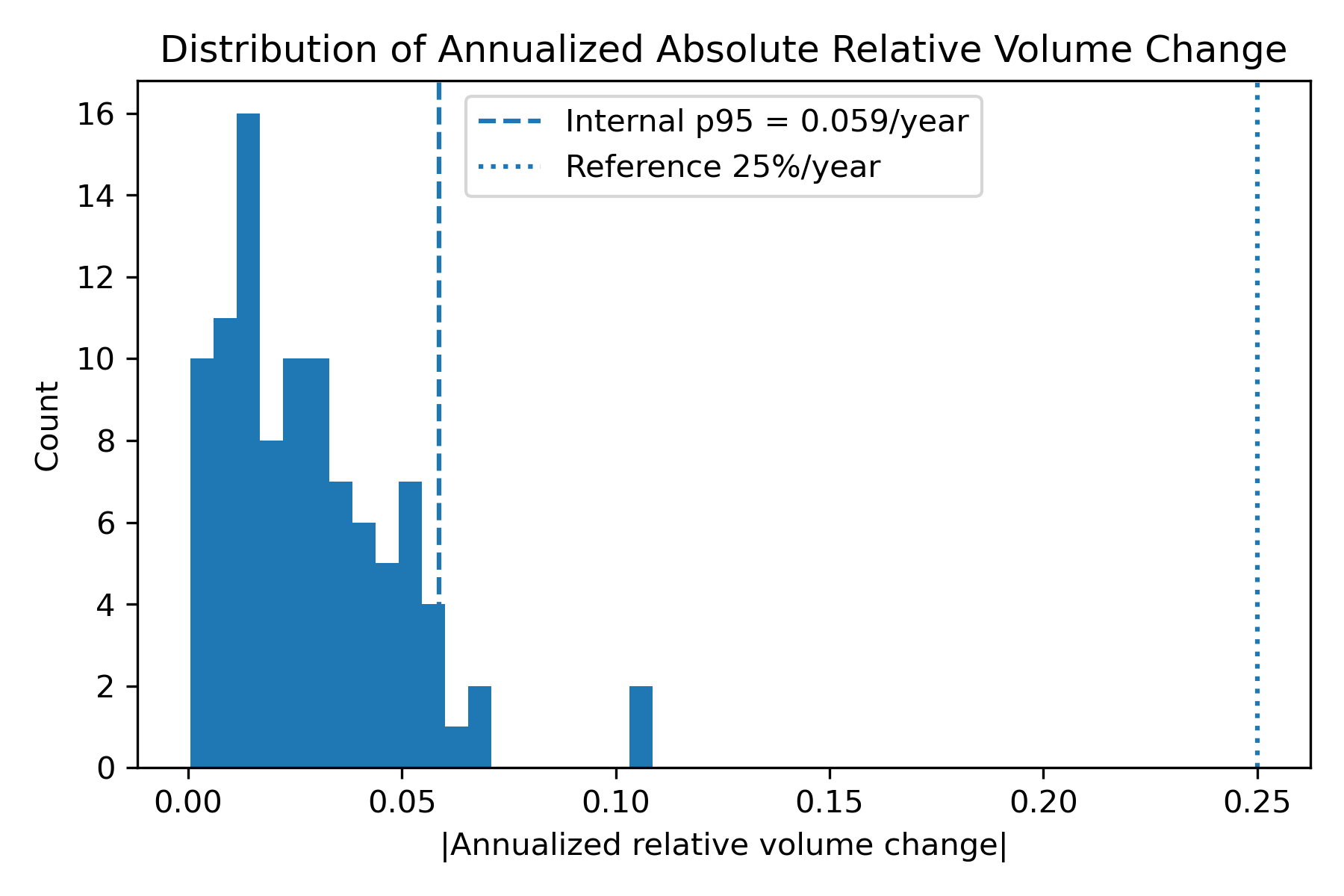


**Figure S3. Histogram of annualised absolute relative volume change.**

*Histogram of* $\mid\Delta V\mid/V_{0}$*per year in the stable cohort. The dashed line indicates the internal 95th percentile (~0.059/year), and the dotted line marks the 25%/year reference threshold.*


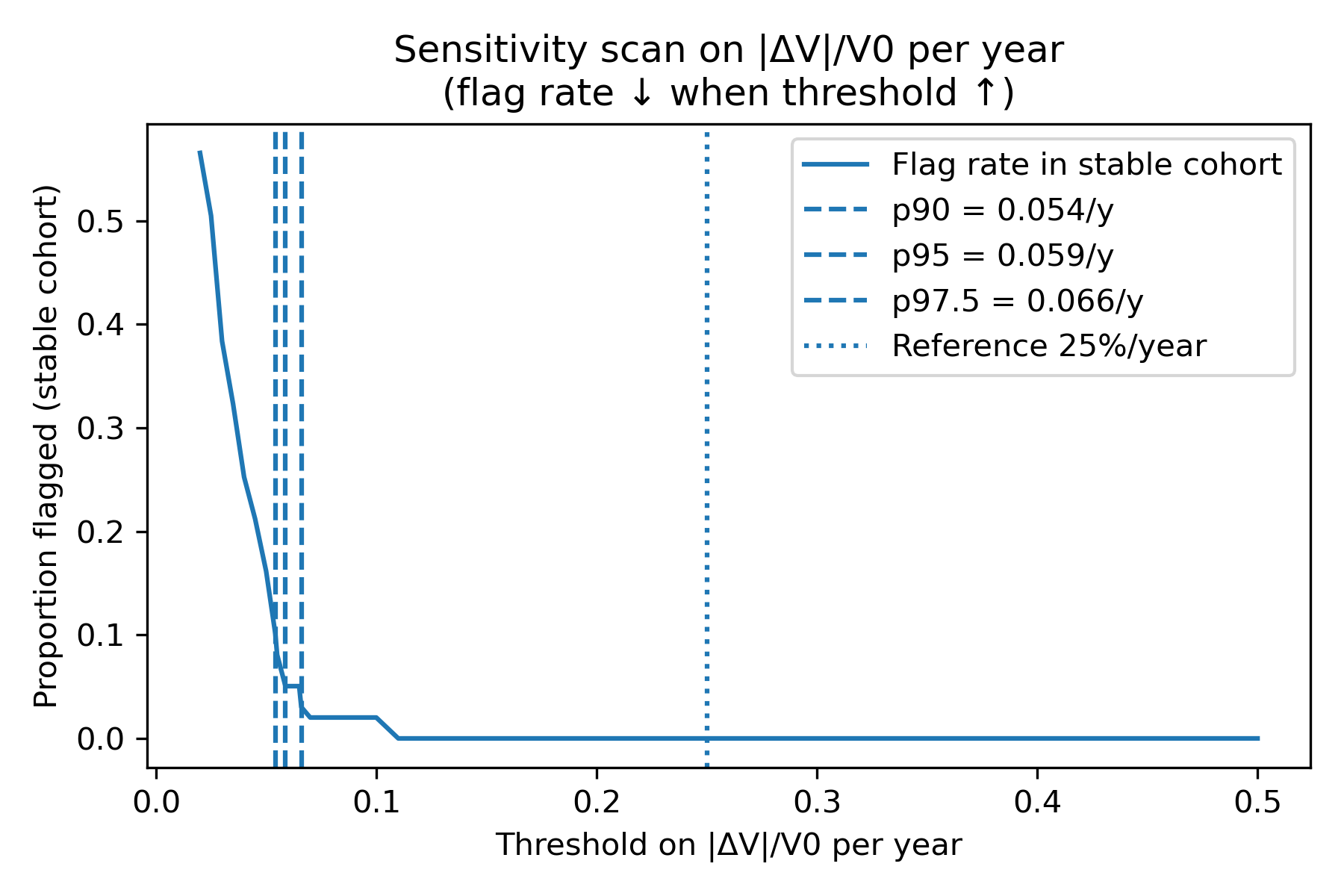


**Figure S4. Threshold scan of flag rate versus annualised absolute relative volume-change threshold.**

*Proportion of nodules in the stable cohort that would be flagged as “growing” (flag rate) as a function of the candidate threshold on* $\mid\Delta V\mid/V_{0}$*per year. Dashed vertical lines indicate the internal 90th, 95th and 97.5th percentile thresholds; the dotted vertical line marks the 25%/year reference threshold. Increasing the threshold decreases the flag rate and increases the internal specificity.*

**S2.1 CT preprocessing and slice-profile equalisation**

All CT examinations were reconstructed using thin slices (≤ 1.5 mm) and lung kernels, in accordance with Fleischner Society technical recommendations. To reduce inter-scanner heterogeneity, we applied a two-step preprocessing pipeline. First, a slice-profile equalisation filter was used to harmonise the slice sensitivity profiles across scanners, approximating a standard target profile as described in previous multicentre radiomics studies. Second, images were resampled to an isotropic voxel spacing of 1.0 × 1.0 × 1.0 mm³ using tri-linear interpolation in SimpleITK, and the corresponding segmentation masks were resampled using nearest-neighbour interpolation to preserve nodule boundaries. Preprocessing parameters and the full YAML configuration file are available upon reasonable request.

**S2.2 Radiomic feature extraction and delta-radiomics pipeline**

Radiomic features were extracted from three-dimensional nodule segmentations at baseline (T0) and follow-up (T1) using PyRadiomics (v3.7.6), in compliance with the Image Biomarker Standardisation Initiative (IBSI) guidelines. Shape features were computed on the original images only, whereas first-order and texture features (grey-level co-occurrence matrix, grey-level run-length matrix, grey-level size-zone matrix, and grey-level dependence matrix) were computed on original, Laplacian-of-Gaussian-filtered, and wavelet-transformed images. Voxel intensities were discretised using a fixed bin width of 25 Hounsfield units, without further normalisation, in order to preserve the physical meaning of Hounsfield units.

For each radiomic feature $f$, we calculated the baseline value $f_{0}$, the follow-up value $f_{1}$, and the corresponding per-day delta-radiomic feature

$$\Delta f_{\text{day}}=\frac{f_{1}-f_{0}}{\text{FollowupDays}},$$

where FollowupDays denotes the number of days between the T0 and T1 scans. Rate-type features, including volumetric and radiomic growth metrics, underwent log(1 + x) transformation prior to modelling to reduce skewness. All feature extraction settings (image types, filters, discretisation, and feature classes) were controlled via a versioned YAML configuration file to ensure full reproducibility.

**S2.3 Derived volumetric, morphologic and attenuation-growth features**

From the volumetric segmentations and linear measurements, we derived a set of simple volumetric, morphologic, and attenuation-growth features that were used as candidate predictors in the clinical-semantic and fusion models. Let $V_{0}$and $V_{1}$denote baseline and follow-up nodule volumes, $L_{0}$and $S_{0}$the baseline long- and short-axis diameters, $L_{1}$and $S_{1}$the corresponding follow-up diameters, $\mu_{0}$and $\mu_{1}$the mean CT attenuation values at baseline and follow-up, and $\Delta t$the scan interval in days. The derived features were defined as follows.

**Volumetric growth metrics**

1.Absolute volume change：$\boldsymbol{\Delta}\boldsymbol{V=}\boldsymbol{V}_{\boldsymbol{1}}\boldsymbol{-}\boldsymbol{V}_{\boldsymbol{0}}\left( \text{m}\text{m}^{\text{3}} \right)$

2.Absolute relative volume change：$\text{pc}\text{t}_{\text{ΔV}}\boldsymbol{=}\frac{\left| \boldsymbol{V}_{\boldsymbol{1}}\boldsymbol{-}\boldsymbol{V}_{\boldsymbol{0}} \right|}{\left| \boldsymbol{V}_{\boldsymbol{0}} \right|}$

3.Absolute volume-change rate (mm^3^/day)**：**$\text{VolRat}\text{e}_{\text{per day}}\boldsymbol{=}\frac{\boldsymbol{V}_{\boldsymbol{1}}\boldsymbol{-}\boldsymbol{V}_{\boldsymbol{0}}}{\boldsymbol{\Delta}\boldsymbol{t}}$

4.Daily absolute relative volume-change rate ：$\boldsymbol{r}_{\mathbf{day}}\boldsymbol{=}\frac{\boldsymbol{\mid}\boldsymbol{V}_{\boldsymbol{1}}\boldsymbol{-}\boldsymbol{V}_{\boldsymbol{0}}\boldsymbol{\mid}}{\boldsymbol{V}_{\boldsymbol{0}}\boldsymbol{\times}\boldsymbol{\Delta}\boldsymbol{t}}$

with the corresponding annualised rate**：**$\boldsymbol{r}_{\mathbf{year}}\boldsymbol{=}\boldsymbol{r}_{\mathbf{day}}\boldsymbol{\times365}$

**Exponential-growth metrics**

When feasible (i.e. $V_{0}>0$, $V_{1}>0$and $V_{1}\neq V_{0}$), we further derived classical exponential-growth metrics:

1.Specific growth rate (SGR, 1/day)：$\text{SG}\text{R}_{\text{per day}}\boldsymbol{=}\frac{\ln\left( \boldsymbol{V}_{\boldsymbol{1}}\boldsymbol{/}\boldsymbol{V}_{\boldsymbol{0}} \right)}{\boldsymbol{\Delta}\boldsymbol{t}}$

2.Volume doubling time (VDT, days)$：\text{VD}\text{T}_{\text{days}}\boldsymbol{=}\frac{\boldsymbol{\Delta}\boldsymbol{t}\boldsymbol{\cdot}\ln\boldsymbol{2}}{\ln\left( \boldsymbol{V}_{\boldsymbol{1}}\boldsymbol{/}\boldsymbol{V}_{\boldsymbol{0}} \right)}$

SGR and VDT were computed using the exponential-growth formulation when valid positive baseline and follow-up volumes and a non-zero follow-up interval were available. Negative SGR or VDT values were retained and indicate interval volume decrease.

**Size, linear-growth, and shape-change metrics**

The equivalent spherical diameter at each time point was defined as$\mathbf{：ESD}\boldsymbol{=}\left( \frac{\boldsymbol{6V}}{\boldsymbol{\pi}} \right)^{\boldsymbol{1}\mathbf{/}\boldsymbol{3}}$

yielding $\mathrm{ESD}_{T0}$and $\mathrm{ESD}_{T1}$. The corresponding ESD growth rate was**：**

$$\text{ESDGrowt}\text{h}_{\text{per day}}\boldsymbol{=}\frac{\text{ES}\text{D}_{\text{T1}}\boldsymbol{-}\text{ES}\text{D}_{\text{T0}}}{\boldsymbol{\Delta}\boldsymbol{t}}$$

If $L_{0},S_{0}$and $L_{1},S_{1}$denote the baseline and follow-up long- and short-axis diameters, we further defined:

Long- and short-axis growth rates：$\text{LongRat}\text{e}_{\text{per day}}\boldsymbol{=}\frac{\boldsymbol{L}_{\boldsymbol{1}}\boldsymbol{-}\boldsymbol{L}_{\boldsymbol{0}}}{\boldsymbol{\Delta}\boldsymbol{t}}$**；**$\text{ShortRat}\text{e}_{\text{per day}}\boldsymbol{=}\frac{\boldsymbol{S}_{\boldsymbol{1}}\boldsymbol{-}\boldsymbol{S}_{\boldsymbol{0}}}{\boldsymbol{\Delta}\boldsymbol{t}}$

Aspect ratios and their change：$\text{Aspec}\text{t}_{\text{T0}}\boldsymbol{=}\frac{\boldsymbol{L}_{\boldsymbol{0}}}{\boldsymbol{S}_{\boldsymbol{0}}}$**；**$\text{Aspec}\text{t}_{\text{T1}}\boldsymbol{=}\frac{\boldsymbol{L}_{\boldsymbol{1}}}{\boldsymbol{S}_{\boldsymbol{1}}}$**；**$\text{Aspec}\text{t}_{\text{T1}}\boldsymbol{=}\frac{\boldsymbol{L}_{\boldsymbol{1}}}{\boldsymbol{S}_{\boldsymbol{1}}}$

As a simple compactness proxy, we approximated the ellipsoid volume at baseline and follow-up as $\boldsymbol{V}_{\mathbf{ellipsoid}\boldsymbol{,T0}}\boldsymbol{=}\frac{\boldsymbol{\pi}}{\boldsymbol{6}}\boldsymbol{L}_{\boldsymbol{0}}\boldsymbol{S}_{\boldsymbol{0}}^{\boldsymbol{2}}\boldsymbol{；}\boldsymbol{V}_{\mathbf{ellipsoid}\boldsymbol{,T1}}\boldsymbol{=}\frac{\boldsymbol{\pi}}{\boldsymbol{6}}\boldsymbol{L}_{\boldsymbol{1}}\boldsymbol{S}_{\boldsymbol{1}}^{\boldsymbol{2}}$

and defined：$\mathbf{CompactnessProxy}_{\boldsymbol{T0}}\boldsymbol{=}\frac{\boldsymbol{V}_{\boldsymbol{0}}}{\boldsymbol{V}_{\mathbf{ellipsoid}\boldsymbol{,T0}}}\boldsymbol{,}\mathbf{CompactnessProxy}_{\boldsymbol{T1}}\boldsymbol{=}\frac{\boldsymbol{V}_{\boldsymbol{1}}}{\boldsymbol{V}_{\mathbf{ellipsoid}\boldsymbol{,T1}}}$

together with their difference

$$\boldsymbol{\Delta CompactnessProxy}\boldsymbol{=}\mathbf{CompactnessProxy}_{\boldsymbol{T1}}\boldsymbol{-}\mathbf{CompactnessProxy}_{\boldsymbol{T0}}\mathbf{.}$$

**Attenuation-change metrics and interaction terms**

For mean CT attenuation within the ROI, denoted by $\mu_{0}$and $\mu_{1}$at baseline and follow-up, we defined:

1.Absolute attenuation change：$\boldsymbol{\Delta}\boldsymbol{\mu=}\boldsymbol{\mu}_{\boldsymbol{1}}\boldsymbol{-}\boldsymbol{\mu}_{\boldsymbol{0}}\boldsymbol{,}$

2.Daily attenuation-change rate：$\mathbf{dMean}_{\mathbf{pe}\mathbf{r}_{\mathbf{d}}\mathbf{ay}}\boldsymbol{=}\frac{\boldsymbol{\Delta}\boldsymbol{\mu}}{\boldsymbol{\Delta}\boldsymbol{t}}\boldsymbol{,}$

3.Relative attenuation change：$\boldsymbol{pct\_dMean}\boldsymbol{=}\frac{\boldsymbol{\mu}_{\boldsymbol{1}}\boldsymbol{-}\boldsymbol{\mu}_{\boldsymbol{0}}}{\boldsymbol{\mid}\boldsymbol{\mu}_{\boldsymbol{0}}\boldsymbol{\mid}}\mathbf{.}$

A small set of clinically motivated interaction terms was also constructed to capture potential synergies between semantic CT features and quantitative growth patterns, for example:

$\text{Spiculation}\boldsymbol{\times}\text{SG}\text{R}_{\text{per day}}$**；**$\text{PleuralIndentation}\boldsymbol{\times}\text{pc}\text{t}_{\text{ΔV}}$**；**$\text{Age}\boldsymbol{\times}\text{IsSmoker}$

For rate-type features, we additionally generated monthly versions by multiplying the per-day rate by 30 (suffix “$per\_month$”) and applied a non-negative $\log(1+x)$transform to reduce skewness. Specifically, for a rate feature $x$, negative values were truncated to zero and

$$\mathbf{lo}\mathbf{g} \boldsymbol{1}\boldsymbol{p}_{\boldsymbol{x}}\boldsymbol{=}\mathbf{l}\mathbf{n} \left( \boldsymbol{1+x} \right)$$

was computed (e.g. ${\log1p\_SGR}_{\mathrm{per}\text{ }\mathrm{day}}$, ${\log1p\_VolRate}_{\mathrm{per}\text{ }\mathrm{day}}$). These derived morphologic and growth features, together with the clinical and semantic variables, were used as candidate predictors in the radiomic-semantic and fusion models.

**S2.4 Radiomic feature harmonisation with ComBat**

To mitigate remaining inter-centre differences in radiomic feature distributions after slice-profile equalisation and resampling, we applied ComBat harmonisation using imaging centre as the batch variable. Before ComBat, non-shape features were standardised to zero mean and unit variance within the training data. ComBat parameters were estimated only within the training folds and then applied to the corresponding validation folds, thereby avoiding information leakage. The external validation cohort was not used to estimate ComBat parameters; for unseen external batches, no extrapolative ComBat adjustment was applied and the original feature values were retained. Shape features and explicitly size-dependent variables were not harmonised.

The impact of ComBat harmonisation was assessed qualitatively by PCA-based visual checks of the radiomic feature matrices before and after harmonisation (Supplementary Fig. S5). Within the training domain, between-centre clustering was visibly reduced after harmonisation. In contrast, the external cohort showed no effective change in the after-ComBat view because unseen external batches were not extrapolatively adjusted. These plots are provided as quality-control visualisations of the harmonisation workflow rather than as a formal measure of transportability.


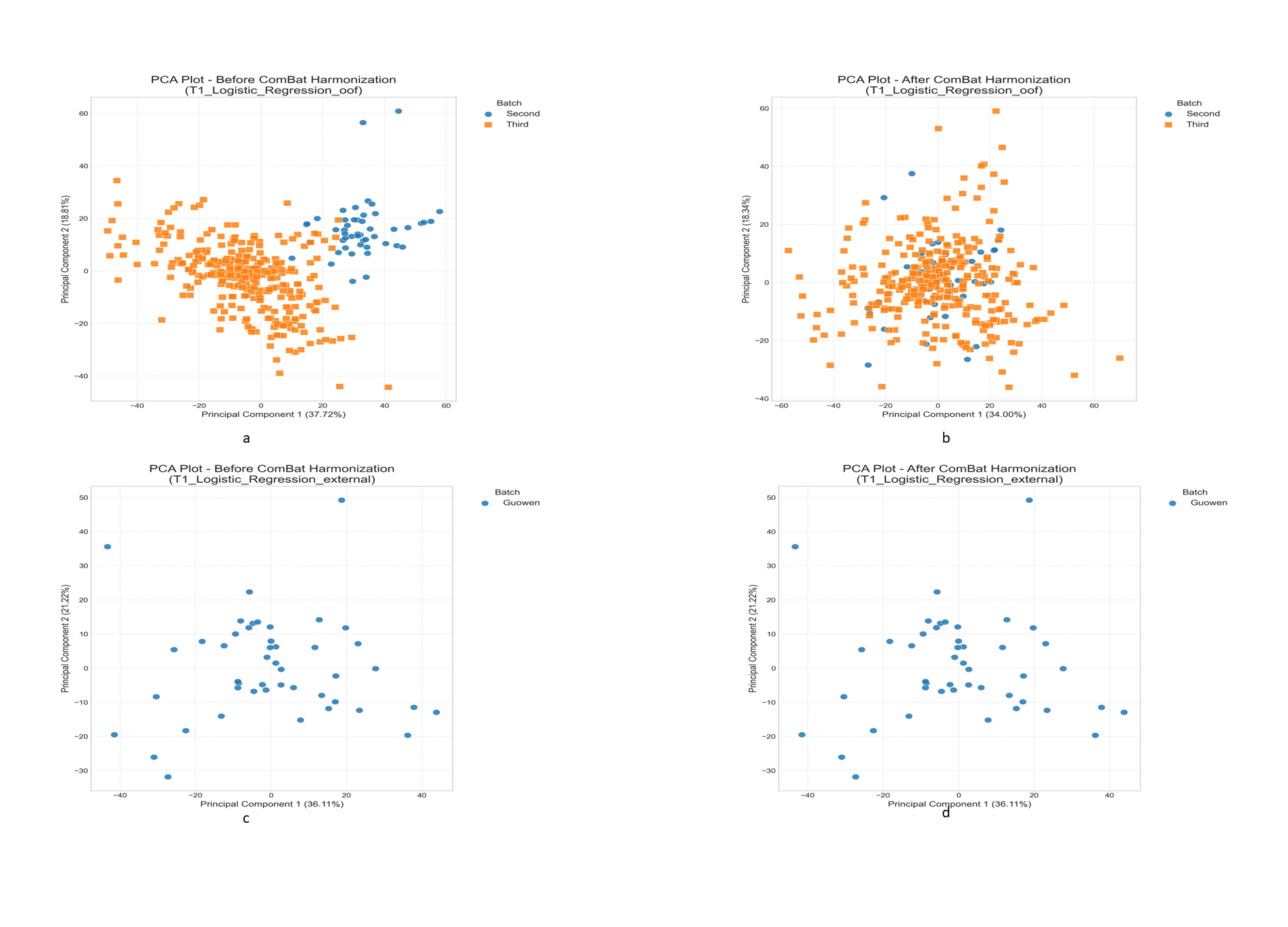


**Supplementary Figure S5. Principal component analysis (PCA) of radiomic feature matrices before and after ComBat harmonisation.**

(a) PCA scatterplot of the training-domain radiomic feature matrix before ComBat harmonisation, with points coloured by imaging centre.

(b) PCA scatterplot of the training-domain radiomic feature matrix after ComBat harmonisation, showing reduced centre-related clustering.

(c) PCA scatterplot of the external radiomic feature matrix before ComBat processing.

(d) PCA scatterplot of the external radiomic feature matrix after ComBat processing. Because the external centre represented an unseen batch relative to the training domain, no extrapolative ComBat adjustment was applied and the original feature values were retained; accordingly, panels (c) and (d) are effectively unchanged. Each point represents one ground-glass nodule.

**S2.5 Detailed model development and feature-selection workflow**

**S2.5.1 Radiomics models and feature selection**

Radiomics models were developed separately for baseline CT (T0), follow-up CT (T1), and delta-radiomics, with delta features defined as the per-day rate of change between T1 and T0 values. For each radiomics dataset, all preprocessing and feature-selection steps were performed strictly within the training portion of each centre-grouped cross-validation fold to avoid information leakage. Missing values were imputed using the median. Batch-effect harmonisation was performed using ComBat, as detailed in Section S2.4.

Feature selection proceeded sequentially through Mann–Whitney U screening with Benjamini–Hochberg false discovery rate correction, embedded L1-penalised logistic regression, and correlation filtering (|r| > 0.70). If no feature passed the false discovery rate threshold within a training fold, a variance-based fallback rule was used to retain a limited set of candidate features for subsequent embedded selection. The final retained features were standardised before classification.

Candidate classifiers included logistic regression, random forest, XGBoost, support vector machine, LightGBM, gradient boosting, AdaBoost, Gaussian naive Bayes, and linear discriminant analysis. Model comparison and hyperparameter optimisation were performed using centre-grouped nested cross-validation, and the best-performing model for each radiomics dataset was selected according to the mean outer-fold area under the receiver operating characteristic curve (AUC). Feature stability was summarised by selection frequency across outer folds.

As an illustrative unimodal example, the correlation structure and selector diagnostics of the final T1 radiomics signature are shown in Supplementary Figs. S6 and S7. A detailed comparison of candidate classifiers across the T0, T1, and delta-radiomics datasets is provided in Supplementary Table S12.

**S2.5.2 Clinical-semantic model**

The clinical-semantic model was constructed from structured clinical, morphologic, and derived longitudinal variables. Candidate variables included age, sex, smoking status, spiculation, lobulation, pleural indentation, initial solid component, solid-component increase, vascular convergence, vacuole sign, air bronchogram, and derived descriptors of size, growth, attenuation change, and shape change.

To reduce redundancy before statistical selection, a predefined family-wise candidate whitelist was applied. Core clinical-semantic candidates comprised Age, Gender, IsSmoker, Spiculation, Lobulation, PleuralIndentation, InitialSolidComponent, SolidComponentIncrease, Vascular, Vacuole, and AirBronchogram. In addition, only one representative candidate was preferentially retained from each predefined feature family according to the following priority orders:

size-related variables (ESD_T0 > LongDiameter > T0_volume_ml > T0_volume_mm3); growth-related variables (VDT_days > log1p_SGR_per_day > SGR_per_day >log1p_VolRate_per_day > VolRate_per_day); attenuation-change variables (dMean_per_day > pct_dMean); and shape-change variables (dCompactnessProxy > Aspect_delta). Predefined interaction terms, including Age × IsSmoker, Spiculation × SGR, and PleuralIndentation × pctdV, were also considered when available. One-hot encoded location and shape variables (Location_* and Shape_*) were retained as candidate predictors and entered the subsequent penalised selection step.

Feature selection was then performed using 5-fold cross-validated L1-penalised logistic regression. Within each fold, variables with non-zero coefficients were recorded as selected features. Variables were subsequently ranked according to selection frequency and mean absolute coefficient magnitude, followed by correlation filtering using an exclusion threshold of |r| > 0.80. Up to six variables were retained. The final classifier was an L2-regularised logistic regression model.

**S2.5.3 Model training, cross-validation, and calibration**

For the clinical-semantic model, model fitting and internal performance estimation were based on centre-aware cross-validation, using grouped out-of-fold (OOF) predicted probabilities for internal assessment. These grouped OOF probabilities were concatenated to obtain approximately unbiased probability estimates for the training cohort. Probabilities were then calibrated using Platt scaling, preferentially fitted on grouped OOF predictions from the training cohort. The final clinical-semantic model was then refitted on the full training cohort using the selected predictors and the same regularisation settings, and the fitted Platt calibrator was applied to the training and external validation cohorts to obtain calibrated probabilities.

For the three radiomics-only models (T0, T1, and delta-radiomics), the original model outputs were treated as raw predicted probabilities and calibrated analogously using Platt scaling fitted on the corresponding training OOF probabilities. The fitted calibrators were then used to generate calibrated probabilities in the training and external validation cohorts. As a representative unimodal example, detailed performance plots for the T1 radiomics model based on OOF predictions in the training cohort and predictions in the independent external validation cohort are shown in Supplementary Fig. S8.

**S2.5.4 Fusion model and three-tier risk stratification**

A second-layer fusion model was constructed using the calibrated output probabilities of the first-layer component models, including T0 radiomics, T1 radiomics, delta-radiomics, and the clinical-semantic model. The fusion classifier was implemented as logistic regression. In the training cohort, 5-fold stratified cross-validation was used to generate OOF fusion probabilities, and model hyperparameters were selected by minimising negative log-loss. Raw OOF fusion probabilities were then calibrated using isotonic regression, and internal fusion discrimination was summarised using the pooled OOF AUC.

Two thresholds ($t_{1}$and $t_{2}$) for three-tier risk stratification were derived from the calibrated OOF probabilities in the training domain. The lower threshold was defined as the highest threshold achieving sensitivity ≥ 95%, thereby prioritising safe rule-out performance. The upper threshold was initially defined as the smallest threshold achieving a positive predictive value of at least 75%; to preserve a clinically meaningful intermediate-risk band and ensure a conservative rule-in boundary, we additionally enforced $t_{2}$≥ $t_{1}$+ 0.05 and $t_{2}$≥ 0.65. The resulting thresholds were then locked and applied unchanged to the training and external-validation cohorts.


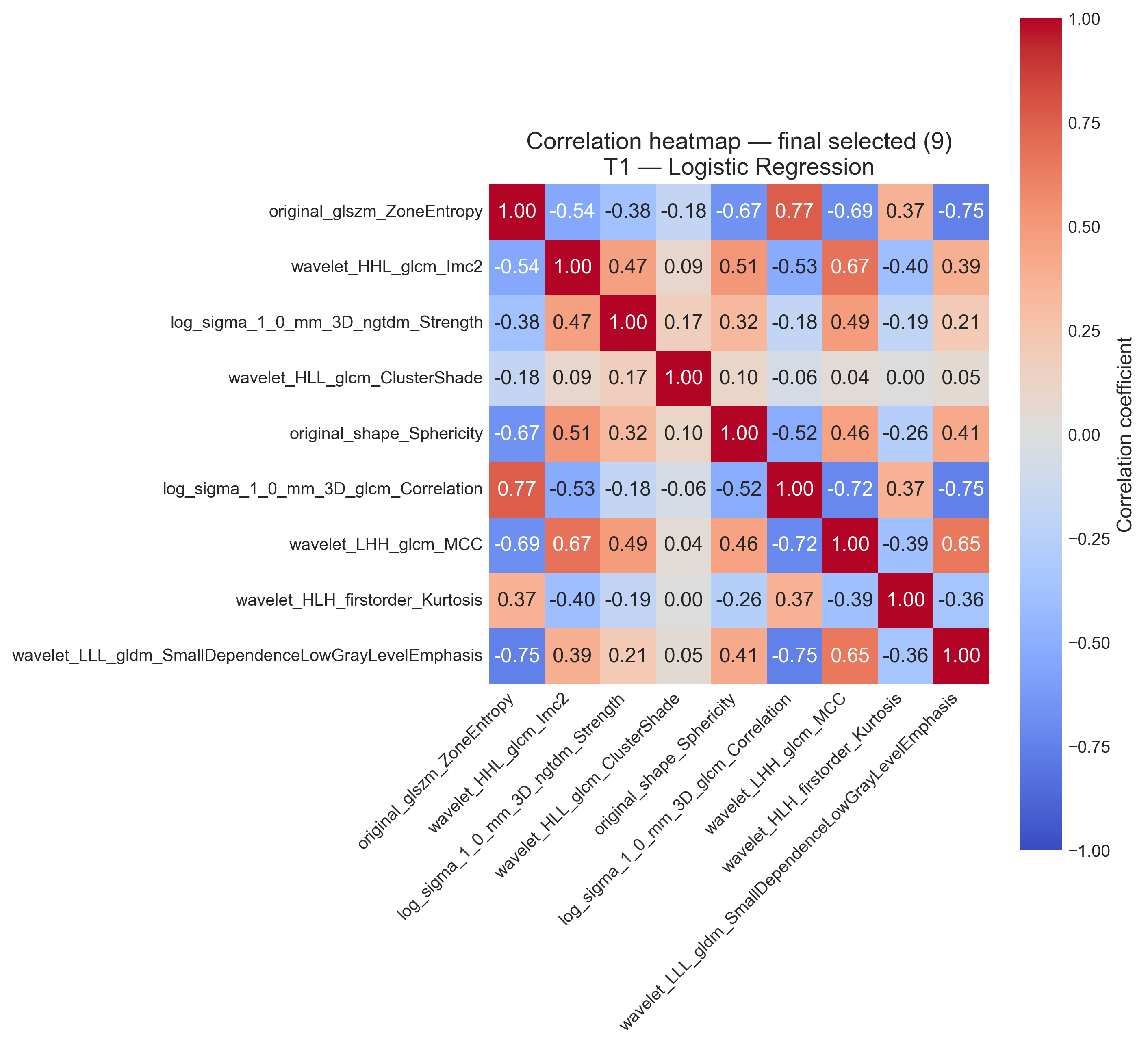


**Supplementary Figure S6. Correlation heatmap of the final selected T1 radiomic features.**

Pairwise Pearson correlation coefficients among the nine radiomic features retained in the final T1 radiomics signature are displayed. Warm colours indicate positive correlations and cool colours indicate negative correlations. Diagonal elements equal 1.0 by definition. Highly redundant features were excluded during sequential feature selection and correlation filtering, and the remaining signature showed only moderate pairwise correlations, helping to reduce multicollinearity in subsequent modelling.**
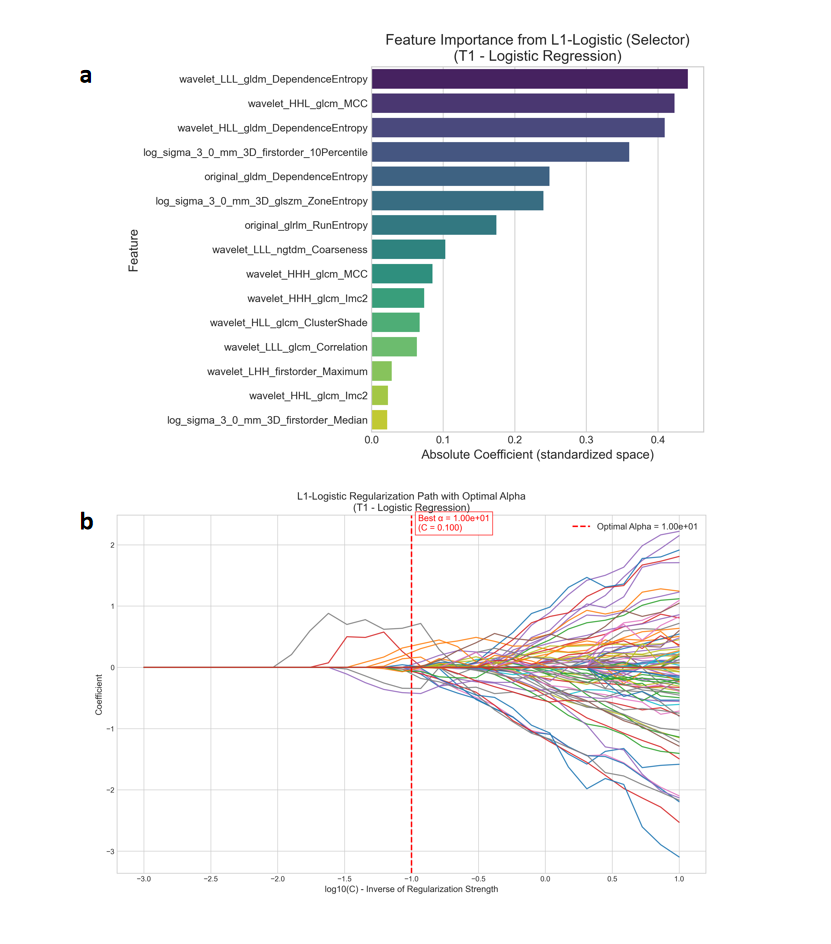
**

**Supplementary Figure S7. Selector diagnostics for the final T1 radiomics signature.**

(a) Absolute standardised coefficients from the L1-penalised logistic selector used during T1 radiomics feature selection. Larger bars indicate greater importance in the selector stage.

(b) L1-regularization path for the T1 radiomics selector. Curves represent coefficient trajectories across values of the regularization parameter, and the dashed vertical line indicates the selected optimal alpha (equivalently, inverse regularization strength 𝐶). Together, these panels illustrate the sparsity-inducing selection behaviour and relative importance of the retained T1 radiomic features.


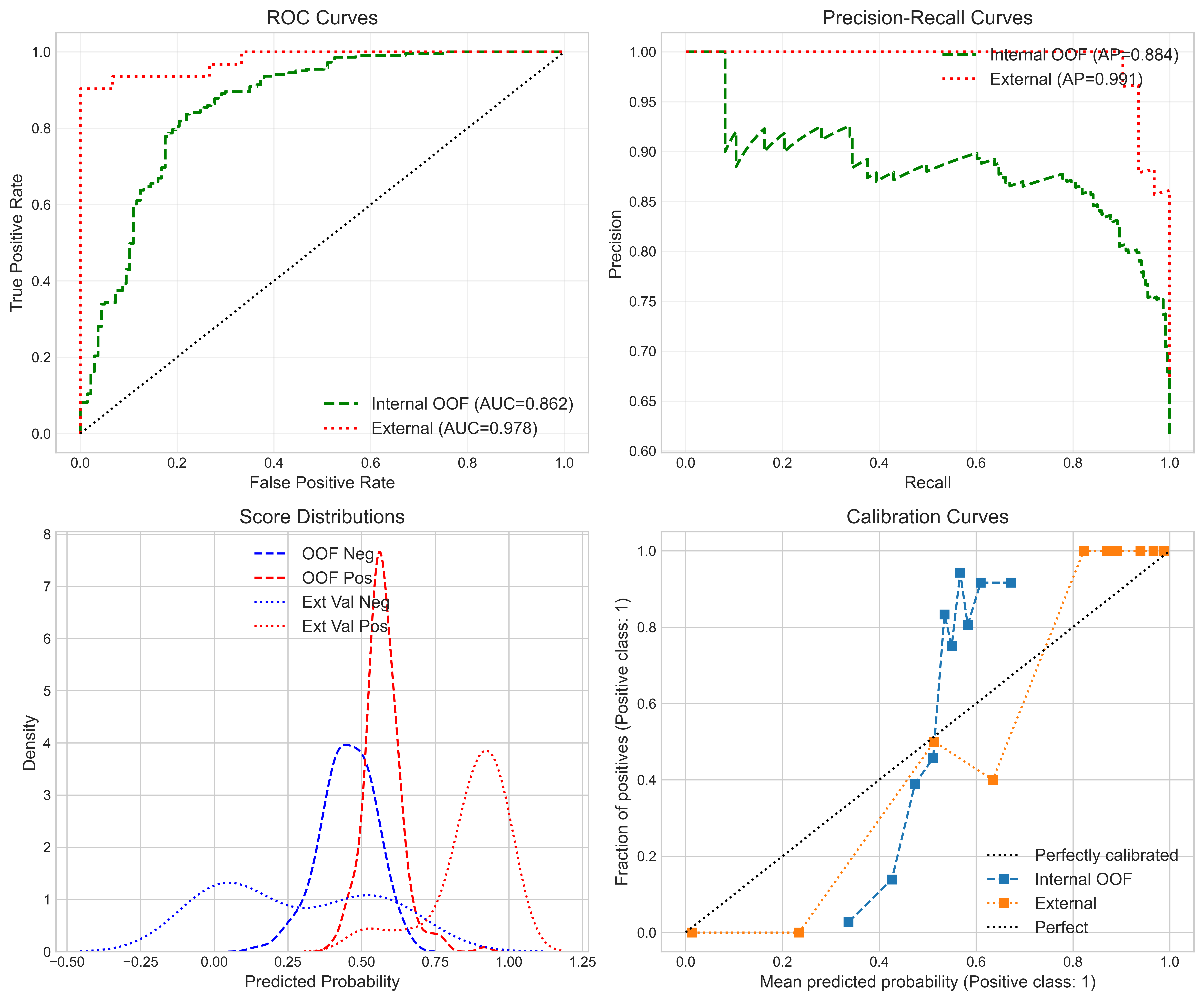


(A) Receiver-operating-characteristic (ROC) curves for the T1 logistic-regression model based on centre-aware internal out-of-fold (OOF) predictions and predictions in the independent external validation cohort.

(B) Precision-recall curves for the same datasets.

(C) Kernel-density estimates of predicted malignancy probabilities, stratified by true outcome (non-invasive vs invasive), illustrating class separation in the internal OOF and external validation datasets.

(D) Calibration curves using decile bins, comparing observed versus predicted event rates. Overall, the T1 model showed good discrimination and acceptable calibration in the internal OOF predictions and in the independent external validation cohort.

**S2.6 Statistical analysis, decision-curve analysis, and guideline benchmarking**

**General performance evaluation and calibration**

Model discrimination was assessed using the area under the receiver-operating-characteristic curve (AUC) for internal out-of-fold (OOF) predictions and for the external validation cohort. For the fusion model, we additionally calculated overall accuracy, sensitivity, specificity, positive predictive value, negative predictive value, and the Matthews correlation coefficient. Probability calibration was evaluated using calibration plots, the Brier score, the expected calibration error (ECE), and the maximum calibration error (MCE), as implemented in the analysis scripts. To aid interpretability, we used SHapley Additive exPlanations (SHAP) to quantify the contribution of individual features and model components to the predicted log-odds of invasive disease. Representative SHAP summary plots for the T1 radiomics model in the internal OOF and external validation datasets are shown in Supplementary Fig. S9.

Clinical utility was evaluated using decision-curve analysis (DCA). For each dataset, we computed the net benefit of the calibrated fusion model across a range of threshold probabilities and compared it with default “treat-all” and “treat-none” strategies. Net benefit was calculated as

$$\mathrm{Net}\text{ }\mathrm{benefit}=\frac{\mathrm{TP}}{N}-\frac{\mathrm{FP}}{N}\times\frac{p_{t}}{1-p_{t}},$$

where $p_{t}$denotes the threshold probability, and $\mathrm{TP}$, $\mathrm{FP}$, and $N$denote the numbers of true positives, false positives, and total patients, respectively.

For three-tier risk stratification, thresholds $t_{1}$and $t_{2}$were derived from calibrated OOF fusion probabilities in the training domain. The lower threshold $t_{1}$(rule-out boundary) was defined as the highest threshold yielding sensitivity $\geq0.95$for invasive lesions. The upper threshold $t_{2}$(rule-in boundary) was initially evaluated against a positive predictive value target of at least 0.75, with additional operating constraints applied as described in the main text. The resulting thresholds were then locked and applied unchanged to the internal OOF and external validation datasets. After fixing $t_{1}$and $t_{2}$, patients were classified into low-, intermediate-, and high-risk bands, and the number of patients and cancer rates in each band were summarised (Supplementary Tables S6B and S9B).

**Guideline benchmarking and decision-analytic comparison**

To benchmark clinical utility against existing management strategies, we compared the calibrated fusion model with Lung-RADS v2022, the 2017 Fleischner recommendations, and a recalibrated Brock-lite model. This analysis was performed at the patient level and was restricted to subsolid nodules, including pure ground-glass nodules (pGGNs) and part-solid nodules (PSNs).

To standardise rule-based and model-based comparisons, baseline lesion size was defined using a hierarchical effective-diameter variable, prioritising directly measured effective spherical diameter, then volume-derived equivalent diameter, then the geometric mean of long- and short-axis diameters, and finally long-axis diameter as a fallback. Growth was conservatively defined as a relative volume increase of at least 25%, a diameter increase of at least 1.5 mm, or the appearance or increase of a solid component.

For Lung-RADS v2022, a conservative implementation for subsolid nodules was used. pGGNs were assigned to low- or intermediate-risk tiers according to baseline size and upgraded to high risk if interval growth or a new/increased solid component was present. PSNs were generally assigned to the intermediate-risk tier and upgraded to high risk when the solid component reached clinically relevant thresholds or when interval growth was observed. For the 2017 Fleischner recommendations, pGGNs were stratified by size and growth, whereas PSNs were conservatively assigned to the high-risk tier. Both guideline systems were operationalised into ordered three-level categories (low, intermediate, and high risk) for direct comparison with the model-based triage framework.

The Brock-lite model was included as a continuous clinical baseline rather than as a formal guideline. It was constructed using available variables aligned with classic malignancy risk modelling, including age, sex, effective diameter, nodule subtype (pGGN vs PSN), upper-lobe location, and spiculation. A logistic-regression pipeline with imputation and standardisation was fitted, and the predicted probabilities were recalibrated using 5-fold cross-validated Platt scaling.

Reclassification performance was assessed using both category-based NRI and binary NRI, and 95% confidence intervals were estimated by 2,000 bootstrap resamples. In the DCA analyses, the high-risk tier was treated as the positive management recommendation for the guideline-based strategies, whereas the fusion model and Brock-lite were evaluated as continuous predictors. Overall, this experiment represents a patient-level decision-analytic comparison of the calibrated fusion model against two guideline-based strategies and one recalibrated clinical risk baseline in a population of pGGNs and PSNs.


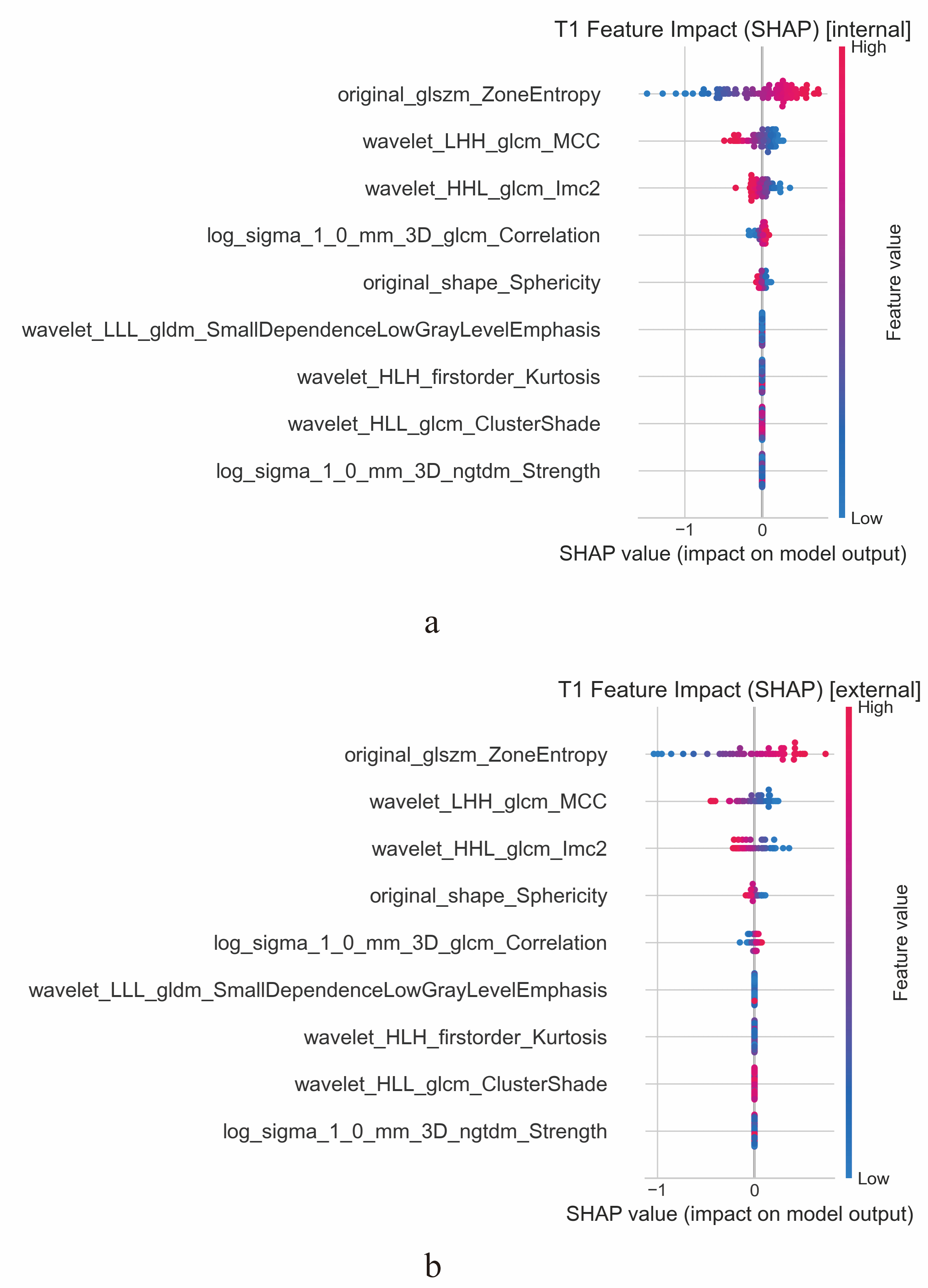


**Supplementary Figure S9. SHAP summary plots for the T1 radiomics model in the internal OOF and external validation datasets.**

(a) SHAP summary plot for the T1 radiomics model based on internal out-of-fold (OOF) predictions. Each point represents one nodule; the x-axis indicates the SHAP value (impact on the predicted log-odds of invasive disease), and colour encodes the corresponding feature value (blue = low, red = high).

(b) Corresponding SHAP summary plot in the independent external validation cohort. Across both datasets, entropy- and shape-related features were among the strongest contributors to model output, with broadly similar directional patterns.

**S2.7 Implementation, software environment, and data/code availability**

All data preprocessing, derivation of morphologic and growth features, radiomic feature extraction, model training, probability calibration, decision-curve analysis, and risk-stratification procedures were implemented using open-source software. The main analysis pipeline was developed in Python (v3.11.3), and selected statistical analyses and figure generation were additionally performed in R (v4.5.1), where appropriate. Radiomic feature extraction was performed using PyRadiomics (v3.7.6). Other major Python libraries included NumPy, pandas, SciPy, scikit-learn, statsmodels, shap, and SimpleITK. Random seeds were fixed where applicable to enhance reproducibility.

The analysis workflow, including preprocessing, derived-feature construction, the clinical-semantic model, probability calibration, the fusion model, risk-score derivation, and three-tier risk stratification, was implemented in annotated scripts and configuration files. Detailed software environment information, including package versions, requirements files, and R session information, can be provided upon reasonable request.

To support reproducibility, the authors are willing to share the analysis code, configuration files, derived feature tables, and figure-generation scripts for academic use upon reasonable request. In addition, de-identified imaging data, segmentation masks/contours, and related metadata may be made available, subject to institutional approval, ethics requirements, and applicable data-protection regulations. Any shared materials will be restricted to data that can be released in a fully de-identified form.

**S2.8 Web-based prototype for SHAP-based interpretability**

To illustrate how the calibrated fusion model may be explored for patient-level risk assessment and transparent model interpretation, we implemented a lightweight web-based prototype based on SHAP (SHapley Additive exPlanations). The prototype runs locally and visualises the calibrated fusion probability, the corresponding 0–100 risk score with three-tier risk bands (low, intermediate, and high), and a SHAP waterfall plot for the four meta-predictor inputs (T0 radiomics, T1 radiomics, delta-radiomics, and the clinical-semantic model). The prototype was implemented in Python with a simple browser-based interface and operates entirely on local hardware without transmitting patient data to external servers.

This prototype is intended solely for research transparency, method illustration, and educational use. It is not a certified medical device or clinical decision-support system and must not be used to guide individual patient management or replace clinical judgement. In addition, its outputs may be affected by deployment-related risks, including distribution shift across scanners, acquisition protocols, and patient populations, as well as automation bias if model explanations are over-interpreted in practice. Any future clinical use would therefore require prospective validation, external testing in broader real-world settings, and preferably a formal decision-impact study or simulation to assess downstream effects on management decisions, false-positive interventions, and patient outcomes.**
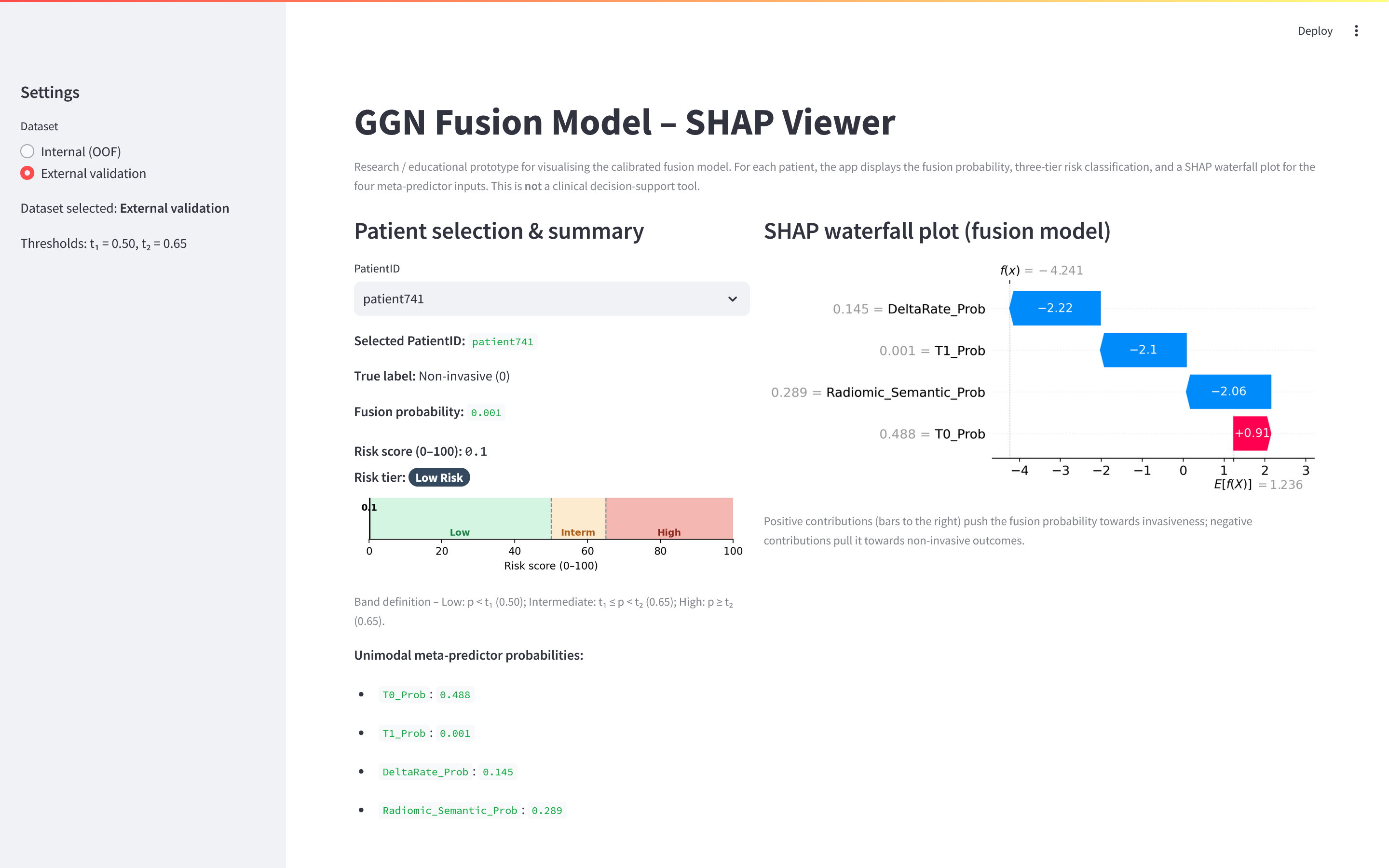
**

**Supplementary Figure S10. Web-based SHAP viewer prototype for the calibrated fusion model.**

Example screenshot of the locally deployed SHAP viewer prototype for the calibrated fusion model. For a selected patient, the interface displays the calibrated fusion probability, the corresponding 0–100 risk score, the assigned three-tier risk classification (low, intermediate, or high risk), and a SHAP waterfall plot showing the contribution of each meta-predictor (T0 radiomics, T1 radiomics, delta-radiomics, and the clinical-semantic model) to the final prediction. The prototype is intended solely for research transparency, method illustration, and educational use, and is not a certified medical device or clinical decision-support system.

**S2.9 Threshold stability and robustness analyses**

To further evaluate the rationale and robustness of the three-tier risk thresholds, we performed supplementary stability and sensitivity analyses based on the calibrated out-of-fold (OOF) fusion probabilities in the training domain. These analyses included bootstrap stability assessment for the rule-out threshold ($t_{1}$) and a decomposed sensitivity analysis for alternative rule-in threshold definitions ($t_{2}$), incorporating bootstrap resampling, fold-to-fold variability, and external robustness analyses.

For the rule-out threshold, $t_{1}$was re-derived in 1,000 bootstrap resamples of the training OOF fusion probabilities using the same method-consistent rule as in the main analysis, namely the highest threshold achieving sensitivity $\geq0.95$for invasive lesions. The resulting distribution was examined to assess the stability of the locked rule-out threshold under training-data perturbation (Supplementary Fig. S11).

For the rule-in threshold, we compared the locked operating threshold used in the main analysis (Scheme A) with alternative rule-in definitions. Scheme B, based on a purely PPV-driven rule, is summarised in Supplementary Table S11 because it yielded a degenerate low threshold and classified a large fraction of cases as high risk. Scheme C applied a constrained data-driven rule combining the PPV target with a minimum separation requirement from $t_{1}$. Bootstrap stability, fold-to-fold variability, and external robustness analyses for these alternative rule-in definitions are summarised in Supplementary Fig. S12. Across these analyses, the rule-out threshold ($t_{1}=0.50$) was held constant, and the sensitivity of external rule-in performance to small perturbations of $t_{2}$around the baseline operating point was evaluated.

**Supplementary Figure S11. Bootstrap distribution of the re-derived rule-out threshold (**$\boldsymbol{t}_{\boldsymbol{1}}$**) under the method-consistent thresholding procedure.**
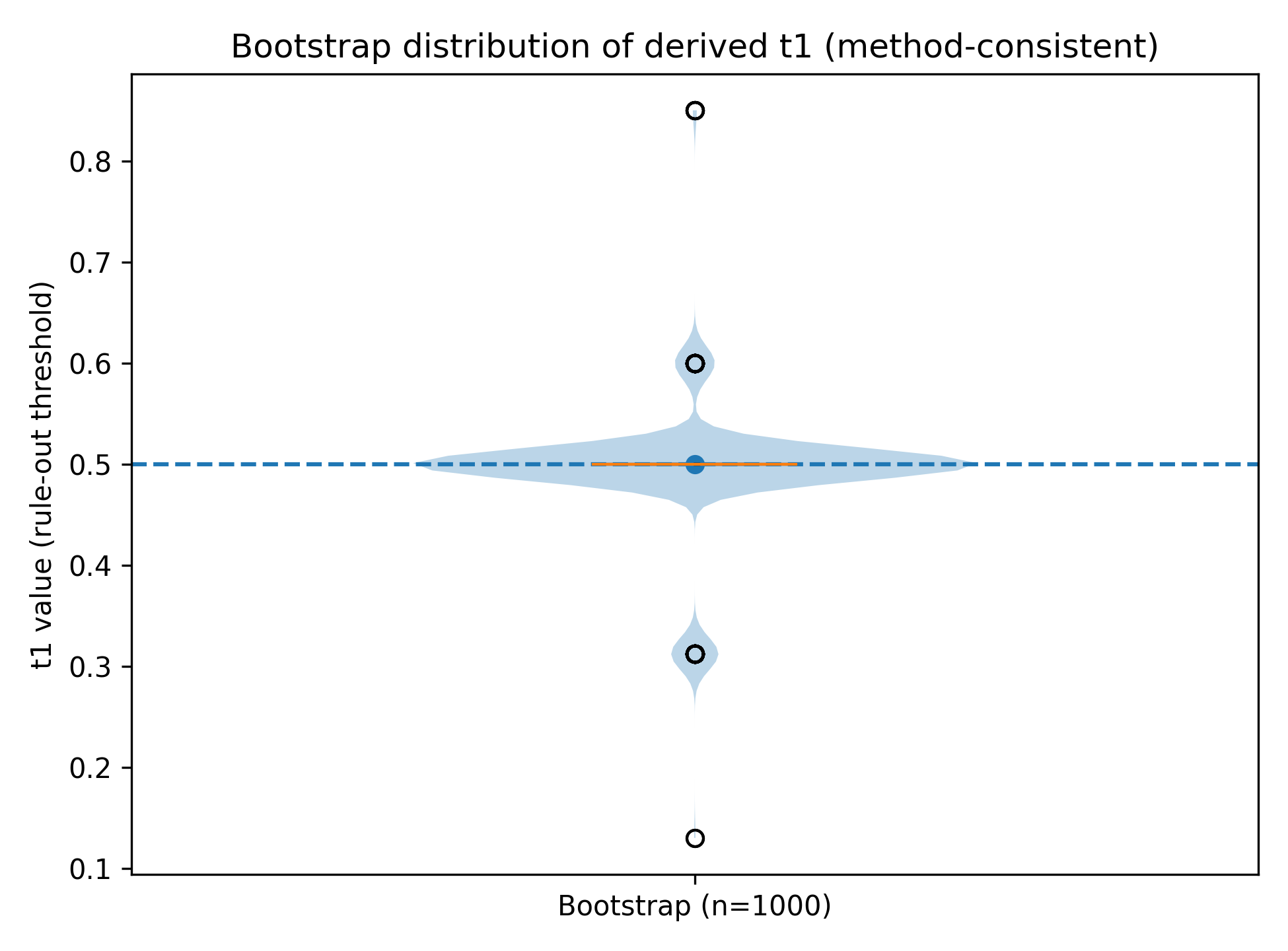


The rule-out threshold was re-derived in 1,000 bootstrap resamples of the training OOF fusion probabilities using the same rule as in the main analysis, namely the highest threshold achieving sensitivity $\geq0.95$for invasive lesions. The distribution was strongly centred around the locked value of 0.50, indicating good stability of the rule-out cutpoint under training-data perturbation.

**Supplementary Figure S12. Decomposed sensitivity analysis of rule-in threshold definitions and robustness of the operating cutpoint.**
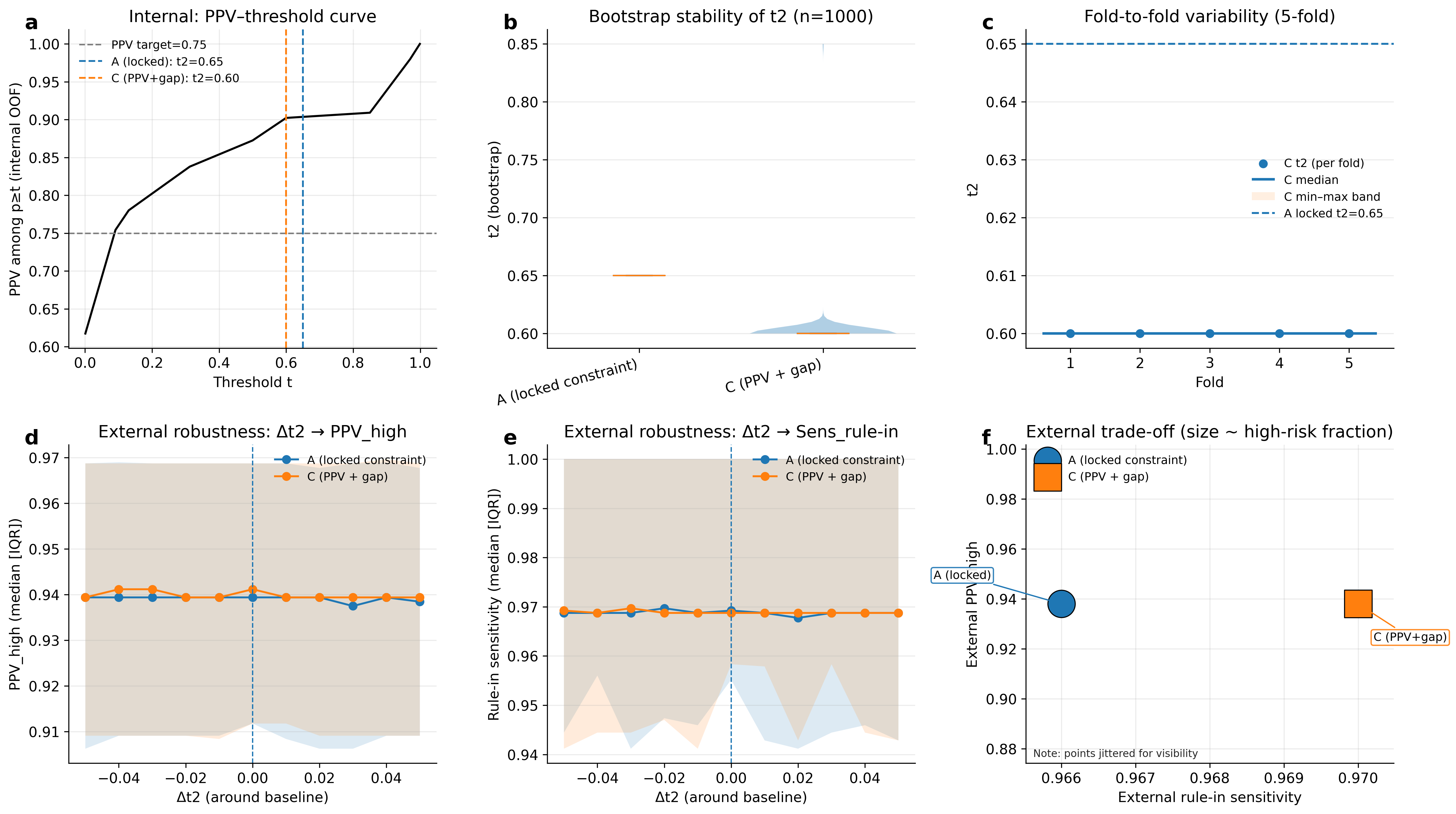


(a) Relationship between the rule-in threshold ($t_{2}$) and the positive predictive value (PPV) among cases classified as high risk ($p\geq t_{2}$) using training OOF fusion probabilities. The horizontal dashed line indicates the PPV target (0.75). Vertical dashed lines indicate the locked operating threshold used in the main analysis (Scheme A: $t_{2}=0.65$) and the constrained data-driven threshold (Scheme C: $t_{2}=0.60$).

(b) Bootstrap stability of $t_{2}$($n=1,000$ resamples), comparing Scheme A (locked operating threshold) and Scheme C (PPV + gap).

(c) Fold-to-fold variability of $t_{2}$under Scheme C across 5-fold resampling; the dashed line marks the locked operating threshold used in Scheme A ($t_{2}=0.65$).

(d–e) External robustness analyses showing that high-risk PPV and rule-in sensitivity are insensitive to small perturbations of $t_{2}$around the baseline operating point.

(f) External trade-off between rule-in sensitivity and PPV; marker size reflects the proportion of cases classified as high risk.

Scheme B (PPV only) is summarised in Supplementary Table S11because it yielded a degenerate low cutpoint, classifying a large fraction of cases as high risk and reducing specificity. The rule-out threshold ($t_{1}=0.50$) was held constant in these analyses.

**S2.10 Fusion ablation analysis**

To assess whether the fusion model could be simplified without material loss of discrimination, we performed an ablation analysis by removing one first-layer branch at a time from the locked full fusion model and re-evaluating the reduced variants in both pooled internal out-of-fold (OOF) predictions and the external validation cohort. The reduced variants were: T1_Delta_ClinicalOnly (without T0 radiomics), T1_ClinicalOnly (without delta-radiomics), and Delta_ClinicalOnly (without T1 radiomics).

Discrimination was summarised using AUC in the internal OOF and external datasets. Performance differences relative to the locked full model were quantified using paired bootstrap $\Delta$AUC (variant minus full model) with 95% confidence intervals and corresponding p values. This analysis was intended to evaluate the marginal contribution of each first-layer branch while preserving the same fusion-model framework.

Overall, removing the T0-radiomics branch produced no material change in discrimination, whereas removing T1 or delta-radiomics resulted in only small decreases in AUC, with confidence intervals crossing zero. These findings support the interpretation that T0 radiomics contributes limited incremental value once explicit clinical-semantic predictors and longitudinal components are already included, while T1 and delta-radiomics provide somewhat greater, although still modest, additional information.


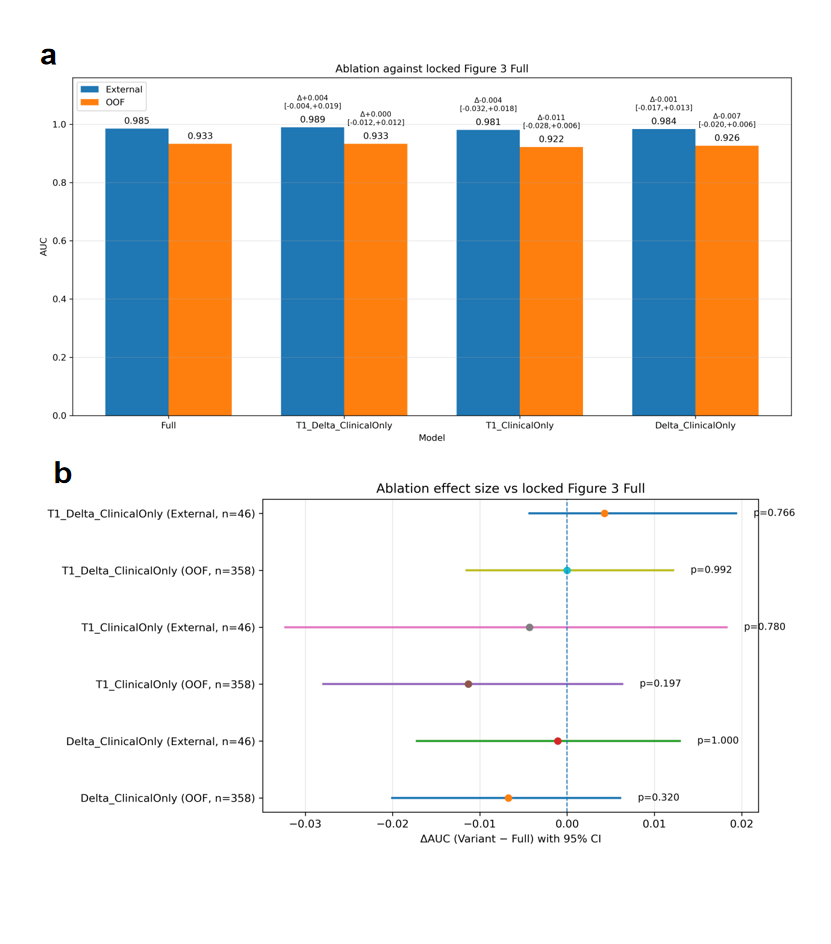


**Supplementary Figure S13. Fusion ablation analysis against the locked full fusion model.**

(A) AUCs of the locked full fusion model and reduced variants in pooled internal out-of-fold (OOF) evaluation and external validation. Reduced variants were constructed by removing one first-layer branch at a time: T1_Delta_ClinicalOnly (without T0 radiomics), T1_ClinicalOnly (without delta-radiomics), and Delta_ClinicalOnly (without T1 radiomics).

(B) Paired bootstrap $\Delta$AUC (variant minus full model) with 95% confidence intervals and p values for each reduced variant relative to the locked full model in internal OOF evaluation and external validation. Positive values indicate improved AUC relative to the full model, whereas negative values indicate reduced AUC.

**Supplementary Table S1. CT acquisition and reconstruction parameters by centre.**

This table summarises the main CT acquisition and reconstruction parameters for thin-section chest CT across the three participating centres.

| Centre | Scanner model | kVp | Pitch | Slice thickness (mm) | Reconstruction kernel | FOV (mm) | Matrix |
| --- | --- | --- | --- | --- | --- | --- | --- |
| Changchun Guowen Hospital | GE Revolution Ace | 120 | 1.0 | 0.6 | LUNG | 380 | 512×512 |
|  | Philips IQon Spectral CT | 120 | 1.2 | 1.0 | YA | 350 | 512×512 |
|  | Philips iCT 256 | 120 | 0.8 | 1.0 | YB | 350 | 512×512 |
|  | Siemens SOMATOM Force | 100 | 1.2 | 1.0 | Bl64d | 361 | 512×512 |
| The Second Hospital of Jilin University | NeuViz Prime | 120 | 0.80 | 1.0 | Lung30 | 350 | 768×768 |
|  | NeuViz Epoch | 120 | 0.80 | 1.0 | Lung10 | 360 | 768×768 |
|  | GE Revolution CT | 140 | 0.99 | 0.625 | HD LUNG | 320 | 512×512 |
|  | Philips iCT 256 | 140 | 0.76 | 1.0 | D | 500 | 1024 × 1024 |
|  | Siemens SOMATOM Force | 120 | 1.2 | 1.5 | Bl57d / Br59d | 346 | 512 × 512 |
| China-Japan Union Hospital of Jilin University | Toshiba Aquilion ONE | 120 | 0.813 | 1.0 | FC52 | 350 | 512×512 |
|  | Toshiba Aquilion Prime | 120 | 0.813 | 1.0 | FC56 | 350 | 512×512 |
|  | GE Revolution CT | 120 | 0.98 | 0.625 | LUNG | 360 | 512×512 |
|  | Philips iCT 256 | 120 | 0.90 | 1.0 | L | 350 | 512×512 |
|  | Siemens SOMATOM Force | 110 | 1.2 | 1.0 | Bl57d | 380 | 512×512 |

**Supplementary Table S2. Inter-observer reproducibility of double-read volumetric, attenuation, and diameter measurements.**

| Measurement | n | ICC (95% CI) |
| --- | --- | --- |
| T0 volume (mm³) | 30 | 0.999 (0.998–1.000) |
| T1 volume (mm³) | 30 | 0.997 (0.993–0.998) |
| T0 mean HU | 30 | 0.991 (0.982–0.996) |
| T1 mean HU | 30 | 0.997 (0.994–0.999) |
| Baseline long diameter (mm) | 30 | 0.994 (0.988–0.997) |
| Baseline short diameter (mm) | 30 | 0.967 (0.933–0.984) |
| Follow-up long diameter (mm) | 30 | 0.989 (0.970–0.995) |
| Follow-up short diameter (mm) | 30 | 0.983 (0.954–0.993) |

**Note:** Inter-observer reproducibility was assessed in a subset of 30 nodules independently reviewed by two radiologists. Intraclass correlation coefficients (ICCs) are reported with 95% confidence intervals for baseline and follow-up volume, mean attenuation, and long- and short-axis diameter measurements.

**Supplementary Table S3. Radiomic feature classes and PyRadiomics settings used in the analysis.**

Supplementary Table S3 summarises the radiomic feature sets and corresponding PyRadiomics settings used in the study, including feature classes, image filters, and intensity discretisation parameters. For each nodule at each time point, we extracted shape features, first-order intensity features, and higher-order texture features, including grey-level co-occurrence matrix, grey-level run-length matrix, grey-level size-zone matrix, grey-level dependence matrix, and neighbourhood grey-tone difference matrix features. Features were extracted from the original images as well as from filtered images generated using wavelet and Laplacian-of-Gaussian filters, in accordance with Image Biomarker Standardisation Initiative recommendations. The complete PyRadiomics parameter file can be provided by the authors upon reasonable request to facilitate reproducibility.

| Image type | Feature class |
| --- | --- |
| Original image | (shape), (first-order), (GLCM, GLRLM, GLSZM, GLDM) |
| (σ = 1.0 mm) / Laplacian-of-Gaussian σ=1.0 mm | (GLCM, GLRLM, GLSZM, GLDM) |
| (σ = 3.0 mm) / Laplacian-of-Gaussian σ=3.0 mm | (GLCM, GLRLM, GLSZM, GLDM) |
| Wavelet-transformed (8 sub-bands) | (GLCM, GLRLM, GLSZM, GLDM) |

**Supplementary Table S4. Final retained radiomic features for each unimodal model and their stability-selection frequencies across resampling schemes.**

| **Model / modality** | **Selected feature** | **Freq (grouped CV; original)** | **Freq (grouped CV; repeated)** | **Freq (random 5-fold×10)** | **Freq (bootstrap)** |
| --- | --- | --- | --- | --- | --- |
| T0 radiomics (LDA) | wavelet_HLH_firstorder_Skewness | 0.5 | 0.5 | 1.0 | 0.8 |
|  | original_shape_Sphericity | 0.5 | 0.5 | 0.88 | 0.6 |
|  | wavelet_HLL_firstorder_Skewness | 0.5 | 0.0 | 0.3 | 0.44 |
|  | original_firstorder_Maximum | 0.5 | 0.5 | 0.04 | 0.08 |
|  | wavelet_HLL_glcm_MCC | 0.5 | 0.0 | 0.08 | 0.06 |
|  | wavelet_HHL_glszm_LargeAreaEmphasis | 0.5 | 0.0 | 0.0 | 0.01 |
|  | log_sigma_1_0_mm_3D_firstorder_TotalEnergy | 0.5 | 0.0 | 0.0 | 0.0 |
| T1 radiomics (Logistic Regression) | wavelet_HLL_glcm_ClusterShade | 0.5 | 0.0 | 0.12 | 0.37 |
|  | log_sigma_1_0_mm_3D_ngtdm_Strength | 0.5 | 0.0 | 0.0 | 0.34 |
|  | original_shape_Sphericity | 0.5 | 0.5 | 0.0 | 0.22 |
|  | wavelet_HLH_firstorder_Kurtosis | 0.5 | 0.5 | 0.02 | 0.2 |
|  | wavelet_HHL_glcm_Imc2 | 0.5 | 0.0 | 0.02 | 0.17 |
|  | log_sigma_1_0_mm_3D_glcm_Correlation | 0.5 | 0.0 | 0.0 | 0.06 |
|  | original_glszm_ZoneEntropy | 0.5 | 0.5 | 0.02 | 0.04 |
|  | wavelet_LHH_glcm_MCC | 0.5 | 0.5 | 0.0 | 0.01 |
|  | wavelet_LLL_gldm_SmallDependenceLowGrayLevelEmphasis | 0.5 | 0.0 | 0.0 | 0.0 |
| DeltaRate radiomics (Random Forest) | wavelet_HHH_gldm_SmallDependenceLowGrayLevelEmphasis | 0.500 | 0.500 | 0.940 | 0.865 |
|  | log_sigma_3_0_mm_3D_glcm_InverseVariance | 0.500 | 0.500 | 0.860 | 0.695 |
|  | wavelet_HLH_gldm_SmallDependenceLowGrayLevelEmphasis | 0.500 | 0.500 | 0.420 | 0.595 |
|  | wavelet_HLL_ngtdm_Contrast | 0.500 | 0.500 | 0.820 | 0.585 |
|  | log_sigma_3_0_mm_3D_glszm_GrayLevelNonUniformity | 0.500 | 0.000 | 0.500 | 0.545 |
|  | wavelet_LLL_glszm_LargeAreaHighGrayLevelEmphasis | 0.500 | 1.000 | 0.460 | 0.505 |
|  | original_shape_Maximum2DDiameterColumn | 0.500 | 0.500 | 0.500 | 0.455 |
|  | wavelet_LHH_ngtdm_Coarseness | 0.500 | 0.500 | 0.520 | 0.445 |
|  | log_sigma_3_0_mm_3D_glcm_Correlation | 0.500 | 0.500 | 0.260 | 0.370 |
|  | original_shape_MinorAxisLength | 0.500 | 0.500 | 0.540 | 0.325 |
|  | wavelet_HLL_firstorder_Kurtosis | 0.500 | 0.500 | 0.280 | 0.325 |
|  | wavelet_LHL_ngtdm_Contrast | 0.500 | 0.500 | 0.260 | 0.295 |
|  | log_sigma_3_0_mm_3D_firstorder_Maximum | 0.500 | 0.500 | 0.080 | 0.275 |
|  | log_sigma_3_0_mm_3D_gldm_DependenceVariance | 0.500 | 0.500 | 0.000 | 0.245 |
|  | wavelet_LLH_gldm_DependenceEntropy | 0.500 | 0.500 | 0.440 | 0.240 |
|  | wavelet_LHH_gldm_LargeDependenceHighGrayLevelEmphasis | 0.500 | 0.000 | 0.280 | 0.240 |
|  | wavelet_HLL_firstorder_Maximum | 0.500 | 0.000 | 0.280 | 0.235 |
|  | log_sigma_1_0_mm_3D_gldm_SmallDependenceLowGrayLevelEmphasis | 0.500 | 0.000 | 0.240 | 0.230 |
|  | wavelet_HHH_glszm_LargeAreaHighGrayLevelEmphasis | 0.500 | 0.000 | 0.240 | 0.220 |
|  | wavelet_HLH_gldm_LargeDependenceHighGrayLevelEmphasis | 0.500 | 0.500 | 0.180 | 0.215 |
|  | wavelet_LLL_glszm_ZoneVariance | 0.500 | 0.000 | 0.280 | 0.210 |
|  | wavelet_LLH_glcm_Imc1 | 0.500 | 0.000 | 0.160 | 0.205 |
|  | wavelet_LLL_gldm_SmallDependenceLowGrayLevelEmphasis | 0.500 | 0.500 | 0.120 | 0.205 |
|  | original_glszm_SizeZoneNonUniformity | 0.500 | 0.500 | 0.280 | 0.190 |
|  | original_ngtdm_Strength | 0.500 | 0.000 | 0.200 | 0.175 |
|  | log_sigma_3_0_mm_3D_glcm_JointAverage | 0.500 | 0.500 | 0.180 | 0.170 |
|  | log_sigma_1_0_mm_3D_gldm_DependenceVariance | 0.500 | 0.000 | 0.040 | 0.150 |
|  | wavelet_HLH_firstorder_Skewness | 0.500 | 0.000 | 0.020 | 0.150 |
|  | wavelet_LHH_glcm_Idn | 0.500 | 0.000 | 0.240 | 0.145 |
|  | wavelet_LLH_glszm_ZoneEntropy | 0.500 | 0.500 | 0.080 | 0.145 |
|  | log_sigma_3_0_mm_3D_glszm_LargeAreaEmphasis | 0.500 | 0.000 | 0.060 | 0.110 |
|  | wavelet_HLL_glszm_ZoneEntropy | 0.500 | 0.000 | 0.180 | 0.100 |
|  | wavelet_LHH_firstorder_Maximum | 0.500 | 0.000 | 0.040 | 0.095 |
|  | wavelet_LLL_glcm_Idmn | 0.500 | 0.000 | 0.020 | 0.055 |
|  | log_sigma_3_0_mm_3D_firstorder_Variance | 0.500 | 0.000 | 0.080 | 0.050 |
|  | wavelet_LHL_firstorder_Energy | 0.500 | 0.000 | 0.080 | 0.040 |
|  | original_shape_Maximum3DDiameter | 0.500 | 0.000 | 0.000 | 0.035 |
|  | wavelet_HHL_glszm_LargeAreaEmphasis | 0.500 | 0.500 | 0.000 | 0.030 |
|  | log_sigma_3_0_mm_3D_glszm_LargeAreaHighGrayLevelEmphasis | 0.500 | 0.000 | 0.000 | 0.015 |
|  | original_glrlm_RunEntropy | 0.500 | 0.000 | 0.000 | 0.005 |
|  | log_sigma_3_0_mm_3D_firstorder_TotalEnergy | 0.500 | 0.000 | 0.020 | 0.000 |
|  | wavelet_LLH_glrlm_ShortRunHighGrayLevelEmphasis | 0.500 | 0.000 | 0.000 | 0.000 |

Frequencies indicate the proportion of resamples in which a feature was retained by the full selection pipeline. All feature-selection steps were performed within the training portion of each centre-grouped cross-validation fold to avoid information leakage. Because the training domain contained only two imaging centres, strict centre-grouped cross-validation yielded relatively coarse frequencies, whereas repeated grouped cross-validation, repeated random 5-fold cross-validation, and bootstrap resampling provided finer-grained stability estimates.

**Supplementary Table S5. Final retained variables of the clinical-semantic model and their selection stability across the original 5-fold cross-validation procedure.**

| Feature | Selection count (out of 5 folds) | Selection frequency | Mean absolute coefficient |
| --- | --- | --- | --- |
| ESD_T0 | 5 | 1.00 | 1.351 |
| SolidComponentIncrease | 5 | 1.00 | 0.467 |
| Vascular | 5 | 1.00 | 0.441 |
| InitialSolidComponent | 5 | 1.00 | 0.201 |
| Spiculation | 5 | 1.00 | 0.134 |
| Spiculation_x_SGR | 4 | 0.80 | 0.136 |

The final clinical-semantic model retained six variables. Selection count and frequency were derived from the original 5-fold feature-selection procedure, and the mean absolute coefficient denotes the average absolute L1-penalised logistic-regression coefficient across folds in which the variable was retained.

**Supplementary Table S6A. Discrimination and calibration metrics for the unimodal and fusion models in the overall cohort (internal out-of-fold and external validation).**

| Dataset | Model | AUC | 95% CI (AUC) | Brier score | Expected calibration error (ECE) | Sensitivity at t₂ = 0.65 | Specificity at t₂ = 0.65 | PPV at t₂ = 0.65 | NPV at t₂ = 0.65 | False positives per 100 non-invasive lesions at t₂ = 0.65 |
| --- | --- | --- | --- | --- | --- | --- | --- | --- | --- | --- |
| Internal (OOF) | T0 radiomics | 0.718 | 0.666–0.773 | 0.206 | 0.035 | 0.643  (0.576-0.706) | 0.715  (0.632-0.789) | 0.785  (0.717-0.842) | 0.554  (0.477-0.628) | 28.5  (21.2-36.5) |
|  | T1 radiomics | 0.862 | 0.815–0.901 | 0.136 | 0.052 | 0.828  (0.772-0.875) | 0.781  (0.702-0.847) | 0.859  (0.805-0.903) | 0.738  (0.658-0.807) | 21.9  (15.3-29.2) |
|  | Delta-radiomics | 0.861 | 0.819–0.903 | 0.132 | 0.048 | 0.792  (0.732-0.843) | 0.781  (0.702-0.847) | 0.854  (0.798-0.899) | 0.699  (0.620-0.771) | 21.9  (14.6-28.5) |
|  | Clinical–semantic | 0.917 | 0.886–0.943 | 0.115 | 0.062 | 0.864  (0.812-0.907) | 0.818  (0.743-0.878) | 0.884  (0.834-0.924) | 0.789  (0.712-0.853) | 18.2  (12.4-24.8) |
|  | Fusion | 0.933 | 0.903–0.958 | 0.085 | 0.014 | 0.905  (0.858-0.940) | 0.839  (0.767-0.897) | 0.901  (0.854-0.937) | 0.846  (0.774-0.902) | 16.1  (10.2-22.6) |
| External validation | T0 radiomics | 0.942 | 0.862–0.995 | 0.152 | 0.157 | 1.000  (0.888-1.000) | 0.667  (0.384-0.882) | 0.861  (0.705-0.953) | 1.000  (0.692-1.000) | 33.3  (13.3-60.0) |
|  | T1 radiomics | 0.978 | 0.940–1.000 | 0.082 | 0.094 | 0.935  (0.786-0.992) | 0.800  (0.519-0.957) | 0.906  (0.750-0.980) | 0.857  (0.572-0.982) | 20.0  (0.0-40.0) |
|  | Delta-radiomics | 0.960 | 0.898–1.000 | 0.089 | 0.145 | 1.000  (0.888-1.000) | 0.800  (0.519-0.957) | 0.912  (0.763-0.981) | 1.000  (0.735-1.000) | 20.0  (0.0-40.0) |
|  | Clinical–semantic | 0.974 | 0.916–1.000 | 0.061 | 0.118 | 0.935  (0.786-0.992) | 0.867  (0.595-0.983) | 0.935  (0.786-0.992) | 0.867  (0.595-0.983) | 13.3  (0.0-33.3) |
|  | Fusion | 0.985 | 0.955–1.000 | 0.042 | 0.060 | 0.968  (0.833-0.999) | 0.867  (0.595-0.983) | 0.938  (0.792-0.992) | 0.929  (0.661-0.998) | 13.3  (0.0-33.3) |

Data are reported as point estimates with 95% confidence intervals where appropriate. AUC denotes the area under the receiver-operating-characteristic curve. Brier score and ECE summarise overall calibration. Sensitivity, specificity, PPV, and NPV are calculated for the binary classification of high risk ($\geq t_{2}$) versus low/intermediate risk ($\left< t_{2} \right.$). False positives per 100 non-invasive lesions are calculated as $(\text{number of non-invasive lesions classified as high risk}\div\text{total number of non-invasive lesions})\times100$.

**Supplementary Table S6B. Three-tier risk stratification with the calibrated fusion model in the overall cohort.**

| Cohort | Risk band | n (patients) | % of cohort | n invasive | n non-invasive | Cancer rate (%) |
| --- | --- | --- | --- | --- | --- | --- |
| Overall – Internal (OOF) | Low Risk | 113 | 31.6 | 7 | 106 | 6.2 |
| Overall – Internal (OOF) | Intermediate Risk | 23 | 6.4 | 14 | 9 | 60.9 |
| Overall – Internal (OOF) | High Risk | 222 | 62.0 | 200 | 22 | 90.1 |
| Overall – External validation | Low Risk | 13 | 28.3 | 0 | 13 | 0.0 |
| Overall – External validation | Intermediate Risk | 1 | 2.2 | 1 | 0 | 100.0 |
| Overall – External validation | High Risk | 32 | 69.6 | 30 | 2 | 93.8 |

The overall cohort includes both pathology-proven lesions and radiologically stable surveillance cases. Cancer rate is defined as the proportion of invasive lesions (MIA/IAC) within each risk band. Percentages are reported to one decimal place where appropriate.

**Supplementary Table S6C. Net reclassification improvement (NRI) for the calibrated fusion model versus guideline-based strategies in the overall cohort.**

| Comparator model | Dataset | Category NRI | 95% CI (Category NRI) | Binary NRI | 95% CI (Binary NRI) |
| --- | --- | --- | --- | --- | --- |
| Fleischner 2017 | External validation | 0.290 | 0.055–0.549 | 0.258 | 0.022–0.519 |
| Fleischner 2017 | Internal (OOF) | 0.193 | 0.086–0.303 | 0.196 | 0.100–0.300 |
| Lung-RADS v2022 | External validation | 0.641 | 0.394–0.839 | 0.609 | 0.361–0.811 |
| Lung-RADS v2022 | Internal (OOF) | 0.364 | 0.254–0.465 | 0.377 | 0.280–0.469 |

Category-based NRI and binary NRI are reported with 95% confidence intervals for comparisons between the fusion model and each guideline-based strategy, separately for the internal out-of-fold and external validation datasets. Positive NRI values indicate improved risk classification by the fusion model relative to the comparator.

**Supplementary Table S7. Baseline characteristics of patients with invasive and non-invasive ground-glass nodules in the pathology-only subset**

|  | **Training cohort** | | | | **Validation cohort** | | | |
| --- | --- | --- | --- | --- | --- | --- | --- | --- |
| **Variable** | **Overall（n=271）** | **Invasive GGN(n=221)** | **Non-invasive GGN(n=50)** | **P value**^1^ | **Overall**  **(n=34)** | **Invasive GGN**  **(n=31)** | **Non-invasive GGN**  **(n=3)** | **P value**^2^ |
| **Age (years), Median [Q1, Q3]** | 57.00 [49.00, 64.00] | 59.00 [51.00, 65.00] | 53.00 [42.00, 61.00] | **<0.001** | 52.12 ± 12.58 | 52.10 ± 13.17 | 52.33 ± 3.21 | 0.94 |
| **Sex, n (%)** |  |  |  | >0.99 |  |  |  | 0.45 |
| Male | 66 (24%) | 54 (24%) | 12 (24%) |  | 6 (18%) | 5 (16%) | 1 (33%) |  |
| Female | 205 (76%) | 167 (76%) | 38 (76%) |  | 28 (82%) | 26 (84%) | 2 (67%) |  |
| **Smoker (Yes), n (%)** | 35 (13%) | 30 (14%) | 5 (10%) | 0.64 | 4 (12%) | 4 (13%) | 0 (0%) | >0.99 |
| **Spiculation (Yes), n (%)** | 93 (34%) | 87 (39%) | 6 (12%) | **<0.001** | 14 (41%) | 14 (45%) | 0 (0%) | 0.25 |
| **Lobulation (Yes), n (%)** | 117 (43%) | 103 (47%) | 14 (28%) | **0.018** | 20 (59%) | 19 (61%) | 1 (33%) | 0.56 |
| **Vacuole (Yes), n (%)** | 100 (37%) | 88 (40%) | 12 (24%) | 0.051 | 14 (41%) | 13 (42%) | 1 (33%) | >0.99 |
| **Air bronchogram (Yes), n (%)** | 125 (46%) | 116 (52%) | 9 (18%) | **<0.001** | 20 (59%) | 20 (65%) | 0 (0%) | 0.061 |
| **Vascular convergence (Yes), n (%)** | 216 (80%) | 186 (84%) | 30 (60%) | **<0.001** | 30 (88%) | 28 (90%) | 2 (67%) | 0.32 |
| **Pleural indentation (Yes), n (%)** | 70 (26%) | 64 (29%) | 6 (12%) | **0.019** | 10 (29%) | 10 (32%) | 0 (0%) | 0.54 |
| **Initial solid component (Yes), n (%)** | 73 (27%) | 71 (32%) | 2 (4.0%) | **<0.001** | 20 (59%) | 19 (61%) | 1 (33%) | 0.56 |
| **Long diameter (mm), Median [Q1, Q3]** | 9.00 [7.00, 11.00] | 9.00 [8.00, 12.00] | 7.00 [5.00, 8.00] | **<0.001** | 10.50 [8.00, 14.00] | 11.00 [9.00, 14.00] | 5.00 [5.00, 6.00] | **0.006** |
| **Short diameter (mm), Median [Q1, Q3]** | 7.00 [5.00, 8.00] | 7.00 [6.00, 9.00] | 5.00 [4.00, 6.00] | **<0.001** | 8.00 [7.00, 10.00] | 8.00 [7.00, 10.00] | 5.00 [5.00, 5.00] | **0.005** |
| **Baseline volume (mL), Median [Q1, Q3]** | 0.35 [0.21, 0.69] | 0.40 [0.24, 0.79] | 0.20 [0.11, 0.32] | **<0.001** | 0.57 [0.35, 1.09] | 0.69 [0.38, 1.11] | 0.18 [0.11, 0.21] | **0.008** |
| **Baseline volume (mm³), Median [Q1, Q3]** | 346.02 [206.10, 692.89] | 399.41 [235.92, 785.00] | 199.44 [113.28, 317.87] | **<0.001** | 572.11 [350.31, 1,086.76] | 692.08 [384.12, 1,114.47] | 175.26 [110.76, 214.00] | **0.008** |
| **Baseline mean CT (HU), Median [Q1, Q3]** | -638.98 [-723.91, -531.37] | -631.66 [-709.14, -531.37] | -686.77 [-777.07, -548.31] | 0.063 | -558.88 [-621.22, -520.88] | -552.13 [-613.07, -513.21] | -644.54 [-645.34, -563.73] | 0.15 |
| **Location, n (%)** |  |  |  | 0.16 |  |  |  | 0.42 |
| Right upper lobe | 115 (42%) | 94 (43%) | 21 (42%) |  | 15 (44%) | 13 (42%) | 2 (67%) |  |
| Right middle lobe | 15 (5.5%) | 13 (5.9%) | 2 (4.0%) |  | 2 (5.9%) | 2 (6.5%) | 0 (0%) |  |
| Right lower lobe | 51 (19%) | 43 (19%) | 8 (16%) |  | 6 (18%) | 6 (19%) | 0 (0%) |  |
| Left upper lobe | 63 (23%) | 54 (24%) | 9 (18%) |  | 8 (24%) | 8 (26%) | 0 (0%) |  |
| Left lower lobe | 27 (10.0%) | 17 (7.7%) | 10 (20%) |  | 3 (8.8%) | 2 (6.5%) | 1 (33%) |  |
| **Nodule shape, n (%)** |  |  |  | **0.004** |  |  |  | **0.020** |
| Regular | 133 (49%) | 99 (45%) | 34 (68%) |  | 10 (29%) | 7 (23%) | 3 (100%) |  |
| Irregular | 138 (51%) | 122 (55%) | 16 (32%) |  | 24 (71%) | 24 (77%) | 0 (0%) |  |

Data are presented as mean ± standard deviation or median [interquartile range] for continuous variables and as n (% of patients) for categorical variables, unless otherwise indicated. The pathology-only subset includes only ground-glass nodules with histological confirmation (AAH, AIS, MIA or IAC). Comparisons between invasive and non-invasive GGNs were performed using Welch’s t test for approximately normally distributed continuous variables, the Wilcoxon rank-sum test for non-normally distributed continuous variables, and the χ² test or Fisher’s exact test (with Monte Carlo simulation where appropriate) for categorical variables. P values are two-sided; bold P values indicate statistical significance at α = 0.05. Abbreviations: GGN, ground-glass nodule; SD, standard deviation; IQR, interquartile range; HU, Hounsfield unit.

**Supplementary Table S8. Follow-up and delta CT characteristics of invasive and non-invasive ground-glass nodules in the pathology-only subset**

|  | **Training cohort** | | | | **Validation cohort** | | | |
| --- | --- | --- | --- | --- | --- | --- | --- | --- |
| **Variable** | **Overall**  **(n=271)** | **Invasive GGN**  **(n=221)** | **Non-invasive GGN**  **(n=50)** | **P value**^1^ | **Overall**  **(n=34)** | **Invasive GGN**  **(n=31)** | **Non-invasive GGN**  **(n=3)** | **P value**^2^ |
| **Follow-up days, Median [Q1, Q3]** | 413.00 [181.00, 892.00] | 413.00 [172.00, 892.00] | 410.50 [201.00, 797.00] | 0.89 | 79.50 [32.00, 399.00] | 71.00 [31.00, 368.00] | 417.00 [42.00, 948.00] | 0.19 |
| **Follow-up volume (mL), Median [Q1, Q3]** | 0.49 [0.29, 0.94] | 0.60 [0.36, 1.14] | 0.22 [0.14, 0.33] | **<0.001** | 0.69 [0.41, 1.37] | 0.72 [0.44, 1.59] | 0.15 [0.14, 0.23] | **0.006** |
| **Follow-up mean CT (HU), Median [Q1, Q3]** | -636.33 [-726.67, -534.76] | -622.53 [-711.96, -538.53] | -693.20 [-771.80, -503.55] | 0.11 | -568.83 [-626.94, -515.87] | -560.17 [-626.94, -509.97] | -611.87 [-637.49, -577.34] | 0.27 |
| **Long diameter at T1 (mm), Median [Q1, Q3]** | 10.00 [8.00, 13.00] | 11.00 [9.00, 13.00] | 7.00 [5.00, 8.00] | **<0.001** | 11.00 [9.00, 14.00] | 11.00 [9.00, 15.00] | 5.00 [5.00, 7.00] | **0.006** |
| **Short diameter at T1 (mm), Median [Q1, Q3]** | 8.00 [6.00, 10.00] | 8.00 [7.00, 11.00] | 5.00 [4.00, 7.00] | **<0.001** | 8.00 [7.00, 10.00] | 9.00 [7.00, 11.00] | 5.00 [5.00, 6.00] | **0.006** |
| **Volume doubling time (days), Median [Q1, Q3]** | 774.08 [230.43, 1,793.25] | 770.55 [309.45, 1,621.34] | 832.40 [-382.73, 2,134.68] | 0.99 | 320.56 [47.27, 1,262.70] | 310.10 [47.27, 1,256.10] | 1,262.70 [-236.39, 10,354.55] | 0.40 |
| **ΔMean HU per day, Median [Q1, Q3]** | 0.00 [-0.07, 0.07] | 0.00 [-0.07, 0.07] | 0.00 [-0.07, 0.04] | 0.64 | -0.02 [-0.11, 0.29] | -0.03 [-0.38, 0.29] | 0.02 [-0.01, 0.78] | 0.40 |
| **ΔCompactness proxy, Median (IQR)** | -0.04 (-0.73 – 0.21) | -0.07 (-0.73 – 0.19) | 0.11 (-0.41 – 0.37) | >0.99 | 0.02 (-0.08 – 0.18) | 0.09 (-0.11 – 0.20) | 0.00 (0.00 – 0.00) | >0.99 |
| **Solid component increase (Yes), n (%)** | 73 (27%) | 70 (32%) | 3 (6.0%) | **<0.001** | 4 (12%) | 4 (13%) | 0 (0%) | >0.99 |

Follow-up and delta metrics are calculated only in patients with at least two CT examinations; missing values therefore reflect absent follow-up. The pathology-only subset includes only ground-glass nodules with histological confirmation (AAH, AIS, MIA or IAC). Data are presented as mean ± standard deviation or median [interquartile range] for continuous variables and as n (% of patients) for categorical variables, unless otherwise stated. Comparisons between invasive and non-invasive GGNs were performed using Welch’s t test, the Wilcoxon rank-sum test, and the χ² test or Fisher’s exact test (with simulated P values where appropriate), as appropriate. P values are two-sided; bold P values indicate statistical significance at α = 0.05. Abbreviations: GGN, ground-glass nodule; SD, standard deviation; IQR, interquartile range; HU, Hounsfield unit; SGR, specific growth rate; VDT, volume doubling time; ESD, equivalent spherical diameter.

**Supplementary Table S9A. Discrimination and calibration metrics for the unimodal and fusion models in the pathology-only subset (internal out-of-fold and external validation).**

| Dataset | Model | AUC | 95% CI (AUC) | Brier score | Expected calibration error (ECE) | Sensitivity at  t₂ = 0.65 | Specificity at  t₂ = 0.65 | PPV at  t₂ = 0.65 | NPV at  t₂ = 0.65 | False positives per 100 |
| --- | --- | --- | --- | --- | --- | --- | --- | --- | --- | --- |
| Internal (OOF) | T0 radiomics | 0.630 | 0.550–0.709 | 0.144 | 0.020 | 1.000  (0.983–1.000) | 0.000  (0.000–0.071) | 0.815  (0.764–0.860) |  | 100.0  (92.9–100.0) |
|  | T1 radiomics | 0.710 | 0.630–0.782 | 0.138 | 0.031 | 0.941  (0.902–0.968) | 0.220  (0.115–0.360) | 0.842  (0.791–0.885) | 0.458 (0.256–0.672) | 78.0  (64.0–88.5) |
|  | Delta-radiomics | 0.570 | 0.472–0.677 | 0.147 | 0.013 | 1.000  (0.983–1.000) | 0.000  (0.000–0.071) | 0.815  (0.764–0.860) |  | 100.0  (92.9–100.0) |
|  | Clinical–semantic | 0.824 | 0.764–0.880 | 0.123 | 0.057 | 0.955  (0.918–0.978) | 0.320  (0.195–0.467) | 0.861  (0.812–0.902) | 0.615 (0.406–0.798) | 68.0  (53.3–80.5) |
|  | Fusion | 0.824 | 0.763–0.882 | 0.114 | 0.022 | 0.914  (0.869–0.947) | 0.480  (0.337–0.626) | 0.886  (0.837–0.924) | 0.558 (0.399–0.709) | 52.0  (37.4–66.3) |
| External validation | T0 radiomics | 0.828 | 0.625–0.969 | 0.081 | 0.026 | 1.000  (0.888–1.000) | 0.000  (0.000–0.708) | 0.912  (0.763–0.981) |  | 100.0  (29.2–100.0) |
|  | T1 radiomics | 0.903 | 0.781–1.000 | 0.080 | 0.140 | 0.968  (0.833–0.999) | 0.000  (0.000–0.708) | 0.909  (0.757–0.981) | 0.000 (0.000–0.975) | 100.0  (29.2–100.0) |
|  | Delta-radiomics | 0.559 | 0.233–1.000 | 0.091 | 0.105 | 1.000  (0.888–1.000) | 0.000  (0.000–0.708) | 0.912  (0.763–0.981) | NA | 100.0  (29.2–100.0) |
|  | Clinical–semantic | 0.892 | 0.636–1.000 | 0.064 | 0.080 | 1.000  (0.888–1.000) | 0.333  (0.008–0.906) | 0.939  (0.798–0.993) | 1.000 (0.025–1.000) | 66.7  (9.4–99.2) |
|  | Fusion | 0.935 | 0.781–1.000 | 0.055 | 0.038 | 1.000  (0.888–1.000) | 0.333  (0.008–0.906) | 0.939 (0.798–0.993) | 1.000 (0.025–1.000) | 66.7  (9.4–99.2) |

NPV is reported as NA when no patients were classified into the corresponding low- or intermediate-risk stratum.

**Supplementary Table S9B. Three-tier risk stratification with the calibrated fusion model in the pathology-only subset.**

| Cohort | Risk band | n (patients) | % of cohort | n invasive | n non-invasive | Cancer rate (%) |
| --- | --- | --- | --- | --- | --- | --- |
| Pathology-only – Internal (OOF) | Low Risk | 14 | 5.2 | 5 | 9 | 35.7 |
|  | Intermediate Risk | 29 | 10.7 | 14 | 15 | 48.3 |
|  | High Risk | 228 | 84.1 | 202 | 26 | 88.6 |
| Pathology-only – External validation | High Risk | 33 | 97.1 | 31 | 2 | 93.9 |
|  | Intermediate Risk | 1 | 2.9 | 0 | 1 | 0.0 |

**Supplementary Table S9C. Net reclassification improvement (NRI) for the calibrated fusion model versus guideline-based strategies in the pathology-only subset.**

| Comparator model | Dataset | Category NRI | 95% CI (Category NRI) | Binary NRI | 95% CI (Binary NRI) |
| --- | --- | --- | --- | --- | --- |
| Fleischner 2017 | External validation | 0.290 | -0.758–1.312 | 0.290 | NA–NA |
|  | Internal (OOF) | -0.065 | -0.276–0.157 | 0.070 | -0.138–0.269 |
| Lung-RADS v2022 | External validation | -0.226 | -0.394–-0.091 | 0.108 | NA–NA |
|  | Internal (OOF) | -0.176 | -0.310–-0.040 | 0.065 | -0.098–0.220 |

NA indicates results that were not estimable because of small sample size or sparse reclassification counts.

**Supplementary Table S10. Bootstrap stability summary of the re-derived rule-out threshold (**$t_{1}$**) under the method-consistent thresholding procedure.**

| Metric | Median | IQR (25th–75th) | 2.5th–97.5th |
| --- | --- | --- | --- |
| Derived $t_{1}$(bootstrap) | 0.5 | 0.500–0.500 | 0.312–0.600 |
| Sensitivity at locked $t_{1}$=0.50 | 0.969 | 0.960–0.975 | 0.942–0.987 |
| NPV in low-risk band (p<$t_{1}$) | 0.939 | 0.922–0.952 | 0.890–0.974 |
| PPV in high-risk band (p≥$t_{2}$) | 0.901 | 0.888–0.914 | 0.861–0.940 |
| Low-risk fraction | 0.316 | 0.299–0.335 | 0.271–0.366 |
| Intermediate-risk fraction | 0.064 | 0.056–0.073 | 0.042–0.089 |
| High-risk fraction | 0.62 | 0.601–0.634 | 0.570–0.668 |

The rule-out threshold $t_{1}$was re-derived in 1,000 bootstrap resamples of the training out-of-fold (OOF) fusion probabilities using the same rule as in the main analysis, namely the highest threshold achieving sensitivity $\geq0.95$for invasive lesions. Summary statistics describe the distribution of the re-derived $t_{1}$values across bootstrap samples.

**Supplementary Table S11. Decomposed sensitivity analysis of alternative rule-in threshold definitions for three-tier risk stratification.**

| Cohort | Metric | Scheme A (locked constraint) | Scheme B (PPV-only) | Scheme C (PPV + gap) |
| --- | --- | --- | --- | --- |
| Internal(OOF) | $t_{1}$/ $t_{2}$ | 0.500 / 0.650 | 0.500 / 0.091 | 0.500 / 0.600 |
|  | High fraction, % | 61.5 (56.3–66.3) | 81.8 (77.5–85.5) | 62.8 (57.7–67.7) |
|  | Low fraction, % | 32.1 (27.5–37.1) | 32.1 (27.5–37.1) | 32.1 (27.5–37.1) |
|  | High PPV, % | 90.9 (86.4–94.0) | 75.4 (70.2–80.0) | 90.2 (85.6–93.5) |
|  | Low NPV, % | 92.2 (85.8–95.8) | 92.2 (85.8–95.8) | 92.2 (85.8–95.8) |
|  | Rule-in specificity, % | 85.4 (78.5–90.3) | 47.4 (39.3–55.8) | 83.9 (76.9–89.1) |
|  | Rule-in sensitivity, % | 90.5 (85.9–93.7) | 100.0 (98.3–100.0) | 91.9 (87.5–94.8) |
|  | Not-low sensitivity, % | 95.9 (92.4–97.8) | 95.9 (92.4–97.8) | 95.9 (92.4–97.8) |
|  | Low n / High n | 115 / 220 | 115 / 293 | 115 / 225 |
|  |  |  |  |  |
| External | $t_{1}$ /$t_{2}$ | 0.500 / 0.650 | 0.500 / 0.091 | 0.500 / 0.600 |
|  | High fraction, % | 69.6 (55.2–80.9) | 80.4 (66.8–89.3) | 69.6 (55.2–80.9) |
|  | Low fraction, % | 28.3 (17.3–42.5) | 28.3 (17.3–42.5) | 28.3 (17.3–42.5) |
|  | High PPV, % | 93.8 (79.9–98.3) | 83.8 (68.9–92.3) | 93.8 (79.9–98.3) |
|  | Low NPV, % | 100.0 (77.2–100.0) | 100.0 (77.2–100.0) | 100.0 (77.2–100.0) |
|  | Rule-in specificity, % | 86.7 (62.1–96.3) | 60.0 (35.7–80.2) | 86.7 (62.1–96.3) |
|  | Rule-in sensitivity, % | 96.8 (83.8–99.4) | 100.0 (89.0–100.0) | 96.8 (83.8–99.4) |
|  | Not-low sensitivity, % | 100.0 (89.0–100.0) | 100.0 (89.0–100.0) | 100.0 (89.0–100.0) |
|  | Low n / High n | 13 / 32 | 13 / 37 | 13 / 32 |

Scheme A used the locked operating threshold applied in the main analysis ($t_{2}$= 0.65). Scheme B used a purely PPV-driven rule-in cutoff defined as the minimum threshold achieving PPV ≥ 0.75 on the training OOF predictions. Scheme C used a constrained data-driven rule-in cutoff requiring both PPV ≥ 0.75 and separation from the rule-out threshold ($t_{2}$ ≥ $t_{1}$ + 0.05). Internal results were computed on training OOF predictions to reduce optimism bias; external results were computed on the independent external validation cohort

**Supplementary Table S12. Performance comparison of nine candidate classifiers for the T0, T1, and delta-radiomics models in centre-grouped nested cross-validation.**

| **Panel A. T0 radiomics model** | | | | | | |
| --- | --- | --- | --- | --- | --- | --- |
| **Classifier** | **Mean outer-fold AUC** | **SD** | **Mean AP** | **Mean F1** | **Mean Recall** | **Mean Precision** |
| **Linear discriminant analysis** | 0.695 | 0.223 | 0.806 | 0.751 | 0.952 | 0.622 |
| Gradient boosting | 0.672 | 0.092 | 0.774 | 0.792 | 0.952 | 0.679 |
| Gaussian naive Bayes | 0.671 | 0.173 | 0.796 | 0.795 | 0.952 | 0.682 |
| Random forest | 0.663 | 0.122 | 0.782 | 0.798 | 0.952 | 0.687 |
| AdaBoost | 0.612 | 0.112 | 0.721 | 0.433 | 0.365 | 0.736 |
| LightGBM | 0.591 | 0.050 | 0.735 | 0.752 | 0.905 | 0.646 |
| XGBoost | 0.523 | 0.023 | 0.696 | 0.783 | 1.000 | 0.644 |
| Logistic regression | 0.503 | 0.003 | 0.677 | 0.746 | 0.929 | 0.626 |
| Support vector machine | 0.472 | 0.039 | 0.623 | 0.768 | 0.952 | 0.646 |
| **Panel B. T1 radiomics model** | | | | | | |
| **Classifier** | **Mean outer-fold AUC** | **SD** | **Mean AP** | **Mean F1** | **Mean Recall** | **Mean Precision** |
| **Logistic regression** | **0.914** | 0.048 | 0.953 | 0.836 | 0.965 | 0.740 |
| Linear discriminant analysis | 0.858 | 0.045 | 0.915 | 0.740 | 0.644 | 0.870 |
| Gaussian naive Bayes | 0.852 | 0.017 | 0.895 | 0.772 | 0.815 | 0.766 |
| Random forest | 0.827 | 0.065 | 0.886 | 0.824 | 0.921 | 0.762 |
| Gradient boosting | 0.809 | 0.068 | 0.863 | 0.686 | 0.574 | 0.855 |
| AdaBoost | 0.805 | 0.056 | 0.862 | 0.727 | 0.679 | 0.814 |
| LightGBM | 0.782 | 0.109 | 0.834 | 0.755 | 0.698 | 0.850 |
| Support vector machine | 0.742 | 0.016 | 0.855 | 0.711 | 0.680 | 0.818 |
| XGBoost | 0.605 | 0.105 | 0.716 | 0.601 | 0.643 | 0.805 |
| **Panel C. Delta-radiomics model** | | | | | | |
| **Classifier** | **Mean outer-fold AUC** | **SD** | **Mean AP** | **Mean F1** | **Mean Recall** | **Mean Precision** |
| **Random forest** | **0.746** | 0.108 | 0.842 | 0.684 | 0.683 | 0.796 |
| Gaussian naive Bayes | 0.716 | 0.177 | 0.784 | 0.831 | 0.919 | 0.764 |
| Linear discriminant analysis | 0.641 | 0.119 | 0.781 | 0.598 | 0.639 | 0.594 |
| Support vector machine | 0.624 | 0.018 | 0.747 | 0.754 | 0.937 | 0.632 |
| AdaBoost | 0.575 | 0.039 | 0.728 | 0.662 | 0.660 | 0.675 |
| LightGBM | 0.574 | 0.006 | 0.727 | 0.582 | 0.524 | 0.811 |
| Gradient boosting | 0.560 | 0.050 | 0.747 | 0.632 | 0.615 | 0.676 |
| XGBoost | 0.557 | 0.057 | 0.682 | 0.770 | 0.929 | 0.665 |
| Logistic regression | 0.477 | 0.023 | 0.615 | 0.695 | 0.786 | 0.658 |

Values are reported as mean outer-fold performance across centre-grouped nested cross-validation. AUC, area under the receiver operating characteristic curve; AP, average precision. The best-performing classifier for each radiomics dataset, selected according to mean outer-fold AUC, is highlighted in bold. Because the training domain contained only two imaging centres, centre-grouped nested cross-validation yielded two outer folds. Accordingly, the mean outer-fold AUC values in this table should be interpreted as strict but coarse centre-aware estimates for classifier screening rather than highly precise rankings of algorithm superiority. Final model performance was further assessed using pooled out-of-fold predictions and independent external validation.
